# Distinct Phyllosphere Microbiome of Wild Tomato Species in Central Peru upon Dysbiosis

**DOI:** 10.1007/s00248-021-01947-w

**Published:** 2022-01-18

**Authors:** Paul Runge, Freddy Ventura, Eric Kemen, Remco Stam

**Affiliations:** 1 Department of Microbial Interactions, IMIT/ZMBP, University of Tübingen, Auf der Morgenstelle 32, 72076 Tübingen, Germany; 2 Department of Plant Microbe Interactions, Max Planck Institute for Plant Breeding Research, Carl-von-Linne-Weg 10, 50829 Köln, Germany; 3 Plant Pathology and Bacteriology, International Potato Centre, Avenida La Molina 1895, La Molina, Lima, Peru; 4 Chair of Phytopathology, TUM School of Life Science, Emil-Ramann-Str. 2, 85354 Freising-Weihenstephan, Germany

**Keywords:** Microbial colonization, Phyllosphere, Microbiome, Tomatoes, Dysbiosis

## Abstract

Plants are colonized by myriads of microbes across kingdoms, which affect host development, fitness, and reproduction. Hence, plant microbiomes have been explored across a broad range of host species, including model organisms, crops, and trees under controlled and natural conditions. Tomato is one of the world’s most important vegetable crops; however, little is known about the microbiota of wild tomato species. To obtain insights into the tomato microbiota occurring in natural environments, we sampled epiphytic microbes from leaves of four tomato species, *Solanum habrochaites*, *S. corneliomulleri*, *S. peruvianum*, and *S. pimpinellifolium*, from two geographical locations within the Lima region of Peru over 2 consecutive years. Here, a high-throughput sequencing approach was applied to investigate microbial compositions including bacteria, fungi, and eukaryotes across tomato species and geographical locations. The phyllosphere microbiome composition varies between hosts and location. Yet, we identified persistent microbes across tomato species that form the tomato microbial core community. In addition, we phenotypically defined healthy and dysbiotic samples and performed a downstream analysis to reveal the impact on microbial community structures. To do so, we compared microbial diversities, unique OTUs, relative abundances of core taxa, and microbial hub taxa, as well as co-occurrence network characteristics in healthy and dysbiotic tomato leaves and found that dysbiosis affects the phyllosphere microbial composition in a host species-dependent manner. Yet, overall, the present data suggests an enrichment of plant-promoting microbial taxa in healthy leaves, whereas numerous microbial taxa containing plant pathogens occurred in dysbiotic leaves.

Concluding, we identify the core phyllosphere microbiome of wild tomato species, and show that the overall phyllosphere microbiome can be impacted by sampling time point, geographical location, host genotype, and plant health. Future studies in these components will help understand the microbial contribution to plant health in natural systems and can be of use in cultivated tomatoes.

**Supplementary Information:**

The online version contains supplementary material available at 10.1007/s00248-021-01947-w.

## Introduction

In the past decade, microbiome studies got a boost through high-throughput sequencing techniques enhancing sequencing quality and depth. Thus, major conclusions about microbial compositions throughout highly variable natural conditions could be drawn from targeted and untargeted sequencing approaches. The majority of plant microbiome studies are related to the model organism *Arabidopsis thaliana* and reveal functional relations between microbial communities and various abiotic and biotic factors. While a fundamental understanding of complex community structures is needed to dissect the relevance of particular microbes in model organisms, further studies on crops and crop wild relatives are of major interest for applied science to secure food production.

### Driving Factors of Phyllosphere Microbiota

Terrestrial plants host distinct microbial communities on various plant organs, superiorly characterized as above-ground (phyllosphere) and below-ground compartments (rhizosphere). Many studies have been published examining the phyllosphere and rhizosphere microbiota [17, 61, 88]. Both plant compartments display unique and overlapping microbial pools. In particular, the phyllosphere inhabits tremendously diverse microorganisms in nature, such as bacteria, archaea, fungi, algae, viruses, and protists (nematodes, protozoa) [2]. The phyllosphere is shaped by leaf surfaces featuring an oligotrophic environment supporting microbe-microbe interactions [78]. Bacteria are the most dominant microbial kingdom on leaves showing 10^4^–10^5^ bacterial cells mm^−2^ [75, 76]. Less is known about yeasts, fungi, and eukaryotes on the phyllosphere, although there is an increasing number of studies profiling eukaryotic microbes across various host species [2, 22, 48, 101]. Colonizers of the phyllosphere originate from various sources, soil, air, rain, and insects (horizontal transmission) or through pollen or seeds (vertical transmission) [20, 36, 102]. In particular, phyllospheric microbes can be colonizer of the surface (epiphytes | phylloplane) and/or cytosolic compartment (endophytes | endosphere). Epiphytes have to cope continuously with changing microhabitat conditions, like light exposure (ultraviolet), high temperatures, and sparse nutrient and water availability and can be rather variable [37, 41, 58, 71, 76]. Besides abiotic stresses, biotic factors incorporate beneficial and pathogenic microbes influencing phyllosphere microbiota assemblies. In this context, the plant immune system indirectly affects microbial consortia by restricting microbial proliferation to secure host health [20],T. [22, 48]. Moreover, hormone cross-talks between abiotic and biotic stress responses have been shown to affect plant fitness maintenance and phyllosphere microbiota [10]. In summary, those findings suggest multidimensional factors shaping microbial communities involving microbe-microbe and microbe-host interactions that in consequence can lead to an alteration in the homeostasis of the plant microbiota. While abiotic factors have been widely studied in manifold host species, the relevance of host genetic factors are less well studied,however, genome-wide association studies have been used to identify certain host factors that impact microbial consortia [9, 48]. Since pathogenic microbes have been identified as major factors in shaping microbial consortia [2], host species or population-specific resistotypes might be of particular interest to examine genotype effects on microbiota.

### Origin of Wild Tomato Species and Their Resistotypes

The tomato clade (*Solanum* section *Lycopersicon*) consists of domesticated tomato, *Solanum lycopersicum,* and wild species native to South America, especially Ecuador, Peru, Chile, and the Galápagos Islands [13, 29, 56, 69, 70]. The wild tomato species occupy overlapping habitats, but interspecific reproduction barriers have been described, which prevent hybridization between most of the closely related species [6]. Multiple populations of wild tomato species, like *S. corneliomulleri*, *S. habrochaites*, *S. pimpinellifolium*, and *S. pennellii* have been described in the Lima region of Peru [6]. Wild tomato species serve as a great genetic pool and have been studied in relation to resistances against major tomato diseases, like bacterial speck, grey mold, early blight, and late blight.

The most prominent bacterial pathogen, *Pseudomonas syringae* pv. tomato race 1 (*Pst*) causes bacterial speck of tomato. Thus, the *Prf*/*Pto* recognition complex is an important model to study effector-triggered immunity in tomatoes [67, 68]. Since co-evolving *Pst* populations overcame *Prf*/*Pto* resistance, wild tomato species like *S. habrochaites* and *S. peruvianum* have been used to map novel quantitative trait loci [8, 90].

In plant pathology, the causal agent of tomato leaf mold *Cladosporium fulvum* has been described as an important model to study binary interaction in tomatoes. Major dominant resistance genes such as Cf-4 and Cf-9 originate from wild tomato species [91]. Homologs can be found in many wild tomato species and show remarkable diversity between populations of certain species, probably to fend off diverse pathogen strains [52, 57].

Early blight disease is one of the most disastrous diseases in tomatoes, infecting aerial parts of the plant during different developmental stages, leading to high economic yield losses across the globe. Species of *Alternaria*, such as *A. alternata*, *A. solani*, *A. linariae*, and *A. tomatophila* have been identified as causal agents of early blight in tomato [1]. On the plant side, multiple resistance sources have been determined in wild tomato species, like *S. habrochaites*, *S. pimpinellifolium, S. peruvianum*, and *S. arcanum* [19, 94]. However, infection assays revealed both resistant and susceptible phenotypes for S*. habrochaites* [94, 103]. Thus, a fully resistant phenotype against early blight is still lacking.

In addition, the foliar oomycete *Phytophthora infestans* (*Pinf*) causes late blight disease in tomatoes. The wild tomato S*. habrochaites* shows resistance to *Pinf*, which is likely steered by polygenes [26]. Also in *S. chilense Pinf* resistance appears to be polygenic and of quantitative nature [53]. In *S. habrochaite*s, multiple quantitative trait loci have been described on chromosomes 4, 5, and 11 [15, 46]. Thus wild tomato species form an exciting and diverse group of species with a large number of natural pathogens.

### Dissecting the Tomato Microbiome

The tomato microbiome and its effect on dominant pathogens have predominantly been studied in cultivated tomatoes, yet several studies included wild relatives. Since the soil microbiome is considered the major inoculum for plant-associated microbes, several recent rhizosphere studies have been published [63, 39]. The most prominent bacterial phyla on tomatoes were Proteobacteria, Bacteroidetes, and Acidobacteria. The majority of rhizosphere microbiota were consistent between tomato cultivars and soil types. In addition to soil types, the rhizosphere microbiota varied significantly in terms of bacterial richness and diversity suggesting a host genotype-dependent microbiome [24]. In contrast to domesticated tomatoes where *Pseudomonas* was observed to be abundant, *Acidovorax*, *Massilia*, and *Rhizobium* were observed on the tomato species *S. pimpinellifolium* [27]. Bacterial profiling of various plant organs of *S. lycopersicum* (cultivar BHN602), such as soil, roots, fruits, and blossom, identified plant compartment and sampling year as the major driver of community compositions [3]. Importantly, recent studies on tomato rhizosphere display that host-associated microbes are crucial for plant health [95]. Transplantation experiments of the rhizosphere microbiome of *S. lycopersicum* cultivar Hawaii 7996 conferred resistance to the soil-borne pathogen *Ralstonia solanacearum* in the susceptible cultivar “Moneymaker” [25, 60]. Responsible for the transmitted resistance phenotype was narrowed down to Flavobacterium (strain TRM1), predominantly occurring in the cultivar Hawaii 7996. Mentioned data suggests a probiotic effect of native microbiomes securing plant health [60].

Seeing the importance of the leaf microbiome, there exists a considerable knowledge gap considering epiphytic microbes on tomato species. Only a few studies on this subject have been published. Toju et al. [92] compared leaf-associated microbiomes of multiple *S. lycopersicum* in the field and showed that bacterial and fungal community compositions remained rather consistent between tomato cultivars. Hence, the sampling site in the field explained most occurring variances. Interestingly, the yeast genus *Hannaella* was frequently found on the tomato cultivar “Ganbarune,” which suggests that host genotypes impact particular microbes on the phyllosphere of tomatoes [92]. A spatial niche within the phyllosphere, the trichome microbiome of *S. lycopersicum* and *S. habrochaites,* has been described by Kusstatscher et al. [59]. While they observed similar bacteria richness on leaves of domesticated and wild tomato species, beta diversities were significantly different between leaves and trichomes of tested tomato genotypes in a glass house study [59].

In this study, we describe the phyllosphere microbiota of several wild tomatoes collected in situ in their natural habitat. Our sampling design consisting of 2 years and multiple semi-overlapping sites allows us to draw the first conclusions on microbiome diversity of wild tomatoes. Moreover, we investigate the effect of dysbiosis in one of the regions.

## Methods

### Sampling Wild Tomato Populations and Field Processing

Wild tomato leaves from natural populations were collected under licenses 391–2017-SERFOR-DGGSPFFS and 451–2018-MINAGRI-SRFOR-DGGSPFFS, for 2 consecutive years from 2018 to 2019 in central Peru from two geographical locations in the Lima region. Detailed sample information are summarized in the Supplementary Table 2. Individual host species were determined visually including *Solanum habrochaites* (2018–2019, Canta), *S. corneliomulleri* (2019, Canta), and *S. pimpinellifolium* and *S. peruvianum* (2018, Yangas). From each plant, healthy and phenotypically dysbiotic leaves (Supplementary Fig. 1) were collected from fully developed, adult plants, and further processed to collect leaf-surface colonizing microbes (epiphytes). Epiphytic compartments were collected in 2-ml screw-cap tubes as described in Agler et al. [2] and directly frozen on dry ice while in the field. Samples were shipped on dry ice to Tübingen (ZMBP, Germany) under SERFOR license 003,535 and stored at − 80 °C until further processing.

### DNA Extraction and Amplicon Library Preparation

Frozen epiphytic fractions from 2018 (108 samples) and 2019 (150 samples) were deep frozen in liquid nitrogen and subjected to bead-beating [2 × 45 s, 6500 rpm] with a custom-beat combination [0.1 mm, 0.5 mm, 2.3 mm zirconium beads]. The resulting powder was used for DNA extraction with a custom protocol, based on phenol–chloroform-isoamyl alcohol followed by magnetic bead clean-up. DNA was quantified with PicoGreen™ and used as input for custom amplicon sequencing libraries. Sequencing libraries were prepared as described in [2]. Bacterial amplicons are targeting 16S rRNA genes V4/V5 regions (compartment of prokaryotic SSU), fungal amplicons are based on ITS2 regions (internal transcribed spacer) and eukaryotic amplicons are targeting 18S rDNA V8/V9 regions (small component of eukaryotic cytoplasmic ribosomes). The complete sample set was sequenced on two runs on an Illumina MiSeq platform using V3 kits (600 cycles). Obtained raw sequencing reads were pre-processed and further downstream analysis was performed as described in 4.2.1.

### Amplicon Quality Processing, Clustering, and Classification

Raw sequencing data were processed using the mothur pipeline (v.1.44.1) [79]. Paired-reads were quality filtered with screen.seqs (parameter = minoverlap = 5, maxambig = 0, maxhomop = 10, minlength = 100, maxlength = 600) and demultiplexed according to their 12 bp barcode-indices. Chimeric sequences were removed using UCHIME and sequences were grouped into operational taxonomic units (OTUs) [34]. Taxonomic assignment was performed based on reference databases for bacterial 16S rRNA genes (Greengenes gg_13_8_99), fungi (Unite, release 02.02.2019), and eukaryotes (PR^2^, v. 4.11.0) [32, 42, 66]. Reference databases were completed with the full phage genomes of PhiX (sequence and taxonomy files), an internal Illumina sequencing standard. Final mothur OTU tables (shared-files) were converted into biom-files and further processed using Qiime2 [14] and in-house R scripts. R packages like qiime2R, phyloseq, and microbiome were implemented for microbial diversity calculations. A beta-dispersion analysis was conducted on Bray–Curtis dissimilarities using rarefied relative abundance OTU tables to calculate sample-to-sample variability for multiple data features (Host × Year × Symptoms). The multivariate homogeneity of group dispersions analysis was conducted within the R package Vegan using “betadisper.” To identify shared and unique OTUs, we grouped samples of Canta tomato species (*S. habrochaites* and *S. corneliomulleri*) according to healthy and dysbiotic leaves. OTUs were filtered and had to be present in at least 10% of all samples. Unique OTUs (taxa) were selected by removing shared and core taxa.

### Microbial Core Calculation

Persistent core microbes across host species and sampling years were calculated using CORE-function in qiime2. Core microbes, represented by operational taxonomic units (OTUs), had to be present in > 85% of all samples to be counted, applied to all amplicons. Multi-sequence alignments using Clustal Omega (v.1.2.4) were conducted, based on representative sequences of each core OTU [82]. Multi-sequence alignments (ClustalW format) of single amplicons were used to calculate rooted phylogenetic trees using iqtree (parameter: iqtree -s *clustalo_output* -st DNA -m TEST -bb 1000 -alrt 1000) [93].

### Network Correlation and Hub Taxa Determination

Cross-kingdom correlation networks were calculated based on Pearson correlation coefficients. Therefore, OTU tables of 16S rRNA, 18S rRNA, and ITS2 amplicons were rarefied on an equal sequencing depth (within each OTU table) and single OTUs were concatenated to summarize taxa on the lowest taxonomic rank (up to genus level). The taxonomy assignment relies on the mothur pipeline output. Pearson correlation coefficients and statistical analysis were performed for the feature Host × Year × Symptom using R. Calculated correlation matrices were further used to infer correlation networks as described in [2]. Network characteristics and layout was determined using Cytoscape (v. 3.8.2) [81]. Multiple network characteristics were implemented within the network layout, such as node degree, betweenness, and closeness centrality. Microbial hub taxa were determined using top 5% values of betweenness and closeness centrality.

### Statistical Analysis

Statistics were performed with either customized scripts using R (pairwise Wilcoxon test, Permanova, Dunn’s test) or qiime2-implemented functions (Kruskal–Wallis test, Permanova).

## Results

### Descriptive Epiphytic Phyllosphere Microbiome of Wild Tomato Species Across Lima Region

#### Host Species and Sampling Year Affects Microbial Richness

In this study, we examined the composition of surface-attached (epiphytic microbes) phyllosphere microbiota of wild tomato species from two geographical origins of the Lima region (western Peru). Samples were collected in 2 consecutive years from 2018 to 2019. Thereby, the wild tomato species *S. corneliomulleri* (2019) and *S. habrochaites* (2018–2019) were collected from multiple high-altitude sampling sites around Canta. In addition, tomato species *S. peruvianum* and *S. pimpinellifolium* were sampled at low-altitude around Yangas in 2018. Our dataset allows the comparison of multiple tomato species, such as *S. habrochaites*, *S. peruvianum*, and *S. pimpinellifolium* within the sampling year 2018. In addition, we were able to compare epiphytic phyllosphere microbiota of *S. habrochaites* and *S. corneliomuller*i in 2019, as well as *S. habrochaites* between 2 consecutive years (2018–2019).

As an overview, we estimated the microbial richness across tomato species in Canta and Yangas. To do so, the samples were rarefied to an equal sequencing depth for bacteria (3780 reads), eukaryotes (3378 reads), and fungi (1552 reads). We observed significant differences in microbial richness of bacteria and eukaryotes (see Supplementary Fig. 2), when comparing samples from Canta and Yangas in 2018, representing multiple tomato species. While Canta represented a lower bacterial diversity (*p* = 3.1e-04) compared to Yangas, we obtained higher diversities of eukaryotes (*p* = 3.9e-05) in Canta. In addition to the impact of geographical locations, we observed a year-to-year variation in Canta from 2018 to 2019, which was significant for bacteria (*p* = 1.68e-03) and eukaryotes (*p* = 5.0e-11). Interestingly, fungal richness remained stable across geographical locations and sampling years.

**Fig. 1:**
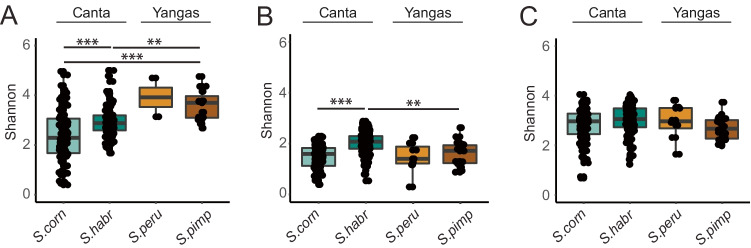
Microbial richness of wild tomato species across the geographical locations Canta and Yangas. Shannon indices are displayed for the following amplicons: **A** bacterial 16S rRNA genes, **B** protists 18S rRNA genes, and **C** fungal ITS2. Statistics: Pairwise-Wilcoxon (p.adjust = Bonferroni), ** =  < 0.01, *** =  < 0.001

Further, we investigated how microbial richness was affected by the host species (see Fig. 1). Interestingly, we observed higher microbial richness in *S. habrochaites* compared to *S. corneliomulleri* for bacteria (*p* = 1.4e-05) and eukaryotes (*p* = 9.5e-04) within the Canta area. In contrast, *S. peruvianum* and *S. pimpinellifolium* from Yangas showed similar microbial diversities for all obtained amplicons. However, the impact of geographical locations on microbial diversities was observed for bacteria and eukaryotes. In detail, we detected significantly lower bacterial richness in *S. corneliomulleri* (Canta) compared to *S. pimpinellifolium* (*p* = 0.0001, Yangas) and lower eukaryotic richness in *S. habrochaites* (Canta) related to *S. pimpinellifolium* (*p* = 0.0069). Those data suggest the geographical origin, as well as the tomato species affecting microbial richness.

### Multivariate Analysis Unveil Distinct Phyllosphere Communities in Host Species

**Fig. 2:**
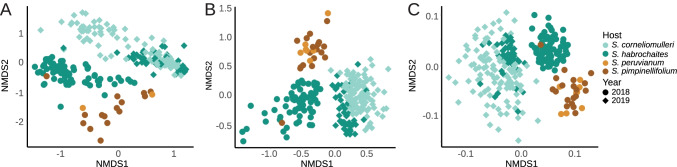
Multivariate analysis of microbiome samples in relation to tomato species and sampling years. Non-metric multidimensional scaling (NMDS) plot on Bray–Curtis dissimilarities showing NMDS1 and NMDS2 represent the first two axes of the two-dimensional ordination. Single dots representing microbiomes of individual leaf samples**. A** bacterial 16S rRNA genes, **B** eukaryotic 18S rRNA genes, and **C** fungal ITS2. Permanova analysis on Bray–Curtis distances (permutations (P) 999) was conducted using Adonis function in R package phyloseq. Samples featuring host & year were highly significant (*p* = 0.001) for all amplicons

We performed a multivariate analysis including non-metric multidimensional scaling (NMDS) and permutational multivariate ANOVA (Permanova, 999 permutations) calculating Bray–Curtis dissimilarities. By comparing phyllosphere microbiota in relation to host species and sampling year (Host × Year), we observed significantly different microbial compositions (*p* = 0.001) across all amplicons (see Fig. 2). These analyses revealed an important impact of the sampling year on the microbial community composition, since phyllosphere microbiomes of *S. habrochaites varied* significantly between the sampling years 2018 and 2019. These year-to-year variations are in line with fluctuating environmental conditions. In addition, we observed a significant effect of tomato species (*p* = 0.001) on the microbiome composition, within Canta, representing *S. corneliomulleri* and *S. habrochaites* (2018)*.* Thus, our findings suggest a year-to-year variation in microbial compositions, as well as host-dependent microbial patterns.

#### Microbial Phyla Abundances Affected Among Tomato Habitats

Fluctuations in microbial compositions dependent on Host × Year were also detectable when we considered the relative abundance on phylum level (see Supplementary Fig. 3A). We performed a statistical analysis using pairwise Wilcoxon tests, based on Host × Year against each taxon on phylum level (see Supplementary Table 1). Especially for bacteria, we identified diverse compositions. Proteobacteria (34–83%), Actinobacteria (6–38%), Firmicutes (1–26%), and Bacteroides (0.23–13%) are the most abundant phyla across all tomato species. In detail, we observed that Proteobacteria were more abundant in *S. habrochaites* (58–83%) and *S. corneliomulleri* (52%) from Canta compared to *S. peruvianum* (38%) and *S. pimpinellifolium* (34%) from Yangas. In contrast, we found higher abundances of Firmicutes in tomato species from Yangas (18–26%) in comparison with Canta species (1–7%). Interestingly, Actinobacteria were highly abundant in Yangas species in 2018 (28–38%), which was not the case for Canta species (7%) within the sampling year, whereas in 2019, Actinobacteria were significantly more abundant in Canta species (36–38%). By inter-species comparison within sampling sites, we observed significantly differences in relative abundance of Actinobacteria in *S. habrochaites* and *S. corneliomulleri* (*p* = 0.026) and *S. peruvianum* and *S. pimpinellifolium* (*p* = 3.7e-08) within 2018. Rare phyla (< 1% rel. Abundance) were concatenated as Others (Acidobacteria, Armatimoadetes, Chlamydiae, Chloroflexi, Elusimicrobia, Fusobacteria, Gemmatimonadetes, Planctomycetes, Spirochaetes, Tenericutes).

Eukaryotic microbial compositions of wild tomato species, obtained from 18S rRNA amplicons, are dominantly composed of Opisthokonta (95–99%) including metazoans and fungi (see Supplementary Fig. 3B). The phylum Stramenopiles, which represents prominent tomato pathogens, like *Phytophthora infestans* was marginally present in Yangas (*S. pimpinellifolium* (0.59%) and *S. peruvianum* (1.8%)) and barely detectable in Canta (*S. habrochaites* (0.08%) and *S. corneliomulleri* (0.0003%)). In addition, we identified Alveolata in *S. habrochaites* (2018, 1.88%). Further eukaryotic phyla, like Ameobozoa, Archaeplastida, Excavata, and Rhizaria are summarized as Others showing < 1% relative abundance.

The relative abundance of fungal taxa was obtained from ITS2 amplicons. Ascomycota (68–87%) is the most prominent fungal phylum across all tomato species, followed by Basidiomycota (8–25%). We also obtained unclassified fungi (0.4–6.2%). Further fungal phyla (Mortierellomycota, Chytridiomycota) are classified as Others showed < 1% relative abundance.

In summary, we observed higher Proteobacteria diversity in tomato species from Canta, whereas Firmicutes were found more abundant in host species from Yangas. Opisthokonta and Ascomycota and Basidiomycota are frequently present in all tomato habitats.

#### Microbial Core Communities and Abundances in Host Species

**Fig. 3:**
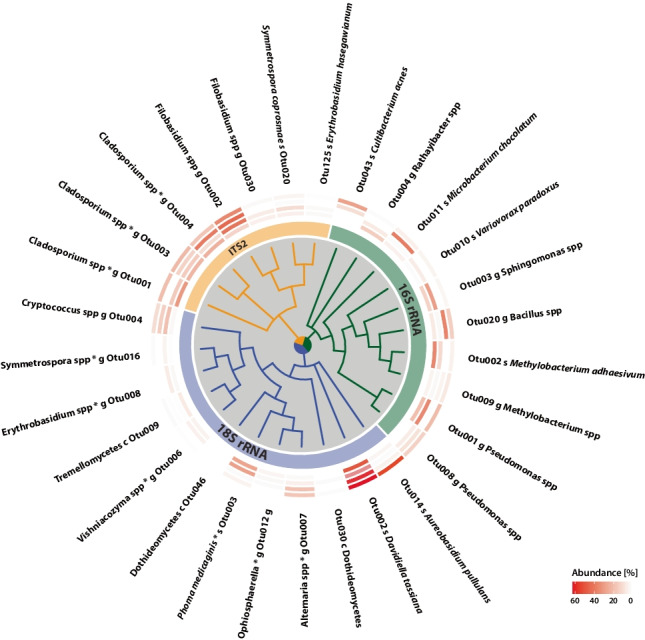
Microbial core OTUs across tomato species and geographical sites. Core microbes are described as $\ge$ 85% occurrence of the full dataset. Phylogenetic trees are created on representative sequences of OTUs [16S rRNA amplicons (green), 18S rRNA amplicons (blue) and ITS2 amplicons (orange)]. Relative abundance of core OTUs are shown for (inner to outer circle) *S. corneliomulleri*, *S. habrochaites*, *S. peruvianum*, and *S. pimpinellifolium*. Taxonomic assignment was performed within mothur pipeline or blast search against nt database (label: *). Taxonomic levels are displayed as class (c), genus (g), or species (s)

We aimed to identify persistent microbial taxa throughout the tomato species across sampling timepoints to verify microbial core communities, presuming their importance in host-microbe and microbe-microbe interactions. We identified 10 bacteria, 7 yeast and 12 fungal OTUs to be present in the core community (> 85% occurrence in all samples of the dataset) (see Fig. 3). We classified 6 Proteobacteria, 3 Actinobacteria, and 1 Firmicutes as bacterial core OTUs. These consisted of diverse bacterial genera known to colonize the phyllosphere, including *Pseudomonas* (Otu001, Otu008), *Sphingomonas* (Otu003), *Methylobacterium*, (Otu002, Otu009), *Rathayibacter* (Otu004), *Variovorax* (Otu010), and Bacillus (Otu020). In addition, we identified *Cultibacterium* (Otu043) and *Microbacterium* (Otu011) as core members, favoring the sampling sites of Yangas.

Further core taxa representing yeasts and fungi were identified using 18S rRNA and ITS2 amplicons. Thus, core OTUs within the kingdom fungi were classified as 10 Ascomycota and 9 Basidiomycota. In detail, fungal-like yeast OTUs are represented by *Erythrobasidium* (18S rRNA: Otu008, ITS2: Otu125), *Symmetrospora* (18S rRNA: Otu016, ITS2: Otu020), *Filobasidium* (ITS2: Otu002, Otu030), and *Vishniacozyma* (18S: Otu006). Fungal OTUs were taxonomically assigned to *Davidiella tassiana* (18S: Otu002), *Aureobasidium pullulans* (18S: Otu014), *Phoma medicaginis* (18S: Otu003), *Ophiosphaerella* (18S: Otu012), and *Cryptococcus* (ITS2: Otu004). In addition, few fungal core OTUs were taxonomically assigned on class level, belonging to Tremellomycetes (18S: Otu009) and Dothideomycetes (18S: Otu030, Otu046). Interestingly, microbial core taxa represent genera that also harbor prominent tomato pathogens, such as *Cladosporium* spp. (syn. *Passalora*) (ITS2: Otu001, Otu003, Otu004) and *Alternaria* spp. (18S: Otu007). *Cladosporium* spp. were highly abundant on both geographical sampling locations Canta and Yangas. The genus *Alternaria*, containing several necrotrophic pathogens for tomato, was predominantly found on *S. corneliomulleri* and *S. habrochaites* in Canta. The fungal plant pathogen *Davidiella tassiana* (18S: Otu002) was highly abundant in Canta and Yangas. We hypothesize that frequently occurring microbial core taxa might have the potential to influence complex microbial communities.

### Impact of Leaf Symptoms on Microbial Community Composition

**Fig. 4:**
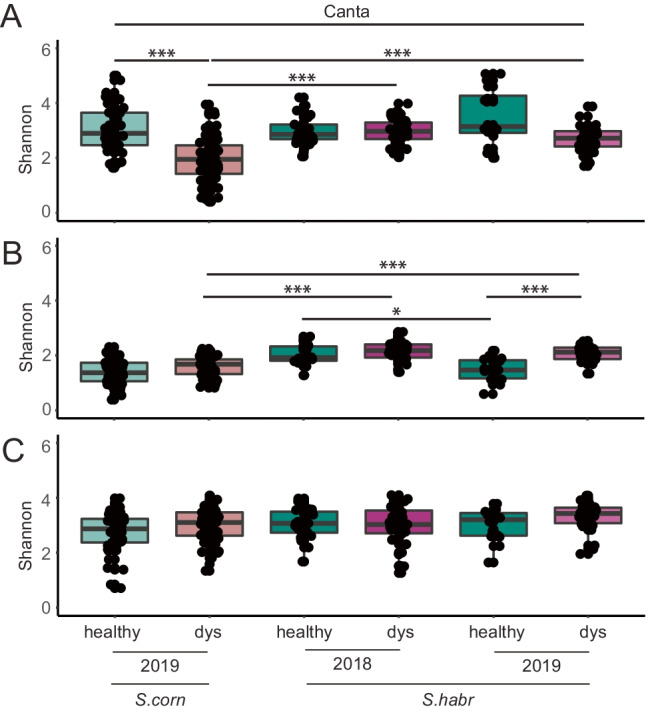
Dysbiosis of wild tomatoes affecting microbial richness in Canta. Shannon indices were calculated for sample groups (Host × Year × Symptom) for **A** bacterial 16S rRNA genes (V4/V5), **B** eukaryotic 18S rRNA genes (V8/V9), and **C** fungal ITS2 amplicons. Pairwise-Wilcoxon-Test was performed with “Bonferroni” *p*-value adjustment using R, *** =  < 0.001

The tomato phyllosphere inhabits various microbes with beneficial, pathogenic, and commensal properties from diverse sources, which colonize throughout the lifespan of its host. Leaves for this study were visually characterized as healthy (green) or dysbiotic (showing growth defects and leaf chlorosis and necrosis) during sample collection (see also Supplementary Fig. 1). Based on this characterization, we aimed to identify microbial key players of epiphytic phyllosphere microbiota that explain detected disease symptoms. We focused on natural populations of tomato species *S. habrochaites* (2018–2019) and *S. corneliomulleri* (2019) from the Canta region, because these samples were best represented in our data set and would allow high-quality analyses. Leaf samples were rarefied to an equal sequencing depth of 8327 reads for bacteria (*n* = 210), 6652 reads for eukaryotes (*n* = 196), and 10,813 reads for fungi (*n* = 215). We first estimated microbial richness (Shannon index that indicates species richness) in healthy and dysbiotic samples for host species from Canta (see Fig. 4). Bacteria showed the highest microbial richness, followed by fungi and eukaryotes.

Richness of bacteria was significantly lower in dysbiotic leaves, compared to healthy leaves of *S. corneliomulleri* (*p* < 0.001), while microbial richness in *S. habrochaites* remained stable independent of plant health in 2 consecutive years. For that reason, the species richness of *S. corneliomulleri* was significantly lower against *S. habrochaites* (*p* < 0.001). In contrast, richness of eukaryotes remained stable in *S. corneliomulleri* (2019) and *S. habrochaites* (2019) in healthy and dysbiotic samples, while dysbiotic leaves from *S. habrochaites* (2018) showed a species enrichment comparing healthy leaves. By comparing species richness of dysbiotic leaves across host species, we identified an enrichment in species diversity in *S. habrochaites* against *S. corneliomulleri* (*p* < 0.001). Interestingly, fungal richness based on ITS2 amplicons remained stable independently of leaf symptoms across host species and years.

From those results, we conclude that the microbial richness was significantly affected upon dysbiosis, which led to either a reduction in species diversity for bacteria or an enhancement for eukaryotes depending on the host species in 2019. The microbial richness of healthy leaves remained stable across host species and sampling years in all targeted profiles (16S rRNA, 18S rRNA, ITS2), while significant differences were obtained for dysbiotic samples across host species and years.

In addition, we conducted a beta-dispersion analysis, considering sample groups of Host × Year × Symptom to identify sample-to-sample variability (see Supplementary Fig. 4). While we observed a higher sample-to-sample variability in *S. corneliomulleri* (2019) for bacteria (*p* < 0.01) and eukaryotes (*p* < 0.001) upon dysbiosis, variability remained unaffected for fungi. Interestingly, we observed a lower distance to centroid for *S. habrochaites* in bacteria (*p* < 0.001) and fungi (*p* < 0.001) upon dysbiosis in the same sampling year. A similar trend of higher distances to centroids for eukaryotes in dysbiotic samples of *S. habrochaites* (2019) was beforehand observed for the microbial richness. While *S. habrochaites* collected in 2018 displayed higher sample-to-sample variabilities for eukaryotes (*p* < 0.01) and fungi (*p* < 0.001) in dysbiotic samples, variability was unaffected for bacteria. Those findings lead to the hypothesis that microbial communities upon dysbiosis become more unstable in *S. corneliomulleri*, whereas they tend to become more stable in *S. habrochaites.*

#### Microbial Consortia Differentiate Between Years and Host Genotype upon Dysbiosis

**Fig. 5:**
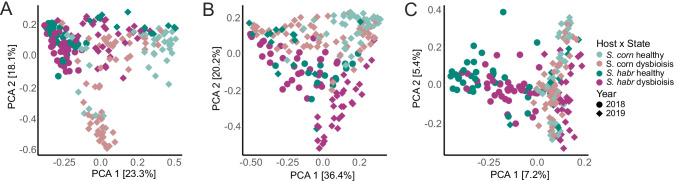
Principal component analysis (PCA) of the taxonomic composition of healthy and dysbiotic samples of *S. habrochaites* and *S. corneliomulleri* from Canta. Shown is the PCA on Bray–Curtis dissimilarities displaying two dimensions PCA 1 and PCA2 in the ordination system. Each point represents one leaf sample colored by their leaf symptoms (healthy, dysbiotic) by host species and shaped by sampling years. Permanova (P 999) was conducted using qiime2; *p* < 0.001

To resolve microbial diversities across leaf samples, we performed a principal component (PCA) analysis using Bray–Curtis dissimilarities grouping samples according to host species, sampling year, and leaf symptoms (Host × Year × Symptom) (see Fig. 5). The explained variance using the first two ordinations, PCA1 and PCA2, within the ordination system reached 40.4% of bacteria, 54.2% of eukaryotes, and 12.6% of fungi. Statistical analysis was performed using permutational multivariate ANOVA (Permanova, permutations (P) 999) calculating Bray–Curtis dissimilarities. Samples of dysbiotic and healthy leaves of *S. habrochaites* and *S. corneliomulleri* remained significantly (*p* = 0.001) different for bacteria, eukaryotes, and fungi across years. Surprisingly, dysbiotic samples of *S. corneliomulleri* (2019) and *S. habrochaites* (2018, 2019) remained significantly different (*p* = 0.001). On the other hand, beta diversities of healthy samples were also significantly different for bacteria, eukaryotes (*p* = 0.001) and fungi (2018: *p* = 0.001, 2019: 0.012). Those results support a host-dependent pathogen perturbation, which affects microbial consortia independently in *S. corneliomulleri* and *S. habrochaites* within one geographical location.

We aimed to identify pathogenic and beneficial microbes guiding distributions of microbial consortia in healthy and dysbiotic samples. To investigate which microbial taxa are shared across *S. habrochaites* and *S. corneliomulleri* upon dysbiosis, we separated operational taxonomic units (OTUs) occurring in healthy and dysbiotic samples. We visualized those findings in Venn-diagrams to obtain unique and shared OTUs in and between four groups (*S. corn.* healthy/dysbiotic; *S. habr.* healthy/dysbiotic) for bacteria (16S rRNA), eukaryotes (18S rRNA), and fungi (ITS2) (see Supplementary Fig. 5). The majority of OTUs were shared across tomato species and independent of leaf symptoms. Thus, 571 OTUs of bacteria, 129 OTUs of eukaryotes, and 267 OTUs of fungi are shared across all Canta samples.

#### Total Read Counts of Core Taxa in Respect to Dysbiosis

We further investigated how microbial core taxa are represented in healthy and dysbiotic samples. Thereby, we summarized all OTUs that belong to a taxonomic level (up to genus level) and compared the total read counts of rarefied samples among leaf symptoms. For that, we implemented the taxonomy assignment revealed from our mothur pipeline. Simplified OTU tables, based on summarized taxa, were created and compared between healthy and dysbiotic samples of each host species.

Our data supports beneficial and pathogenic microbial core taxa in healthy and dysbiotic samples across Canta samples. Thereby, bacterial genera like *Bacillus* (*p* =  < 0.001) and *Pseudomonas* (2019, *p* =  < 0.001) were mainly more abundant in healthy tomato leaf samples. On the other hand, dysbiotic samples showed increased total read counts of *Methylobacterium* (*S. habrochaites* 2019, *p* =  < 0.001), *Microbacterium* (*S. habrochaites* 2018 and *S. corneliomulleri* 2019, *p* =  < 0.001), and *Rathayibacter* (*S. habrochaites*, *p* =  < 0.001) across tomato species. Interestingly, *Variovorax* was predominantly present in dysbiotic samples of *S. habrochaites* (2018–2019). In contrast to bacterial genera, yeast and fungal genera and classes displayed more inconsistency across tomato species and sampling years. While in *S. habrochaites* (2018), yeasts like *Cryptococcus* and *Symmetrospora* and fungal genera *Davidiella* and *Filobasidium* were dominated in healthy samples, fungal classes like Dothideomycetes (18S rRNA and ITS2) and Tremellomycetes were numerously found in dysbiotic leaves. Contrasting, *S. habrochaites* (2019) showed trends of predominantly genera like *Cryptococcus*, *Symmetrospora*, *Davidiella*, and *Filobasidium* in dysbiotic samples. Statistical tests on total read counts using pairwise Wilcoxon test (*p*-value adjustment “Bonferroni”) revealed an enhancement of Tremellomycetes (*p* =  < 0.001) and *Erythrobasidium* (*p* =  < 0.01) in dysbiotic samples of *S. habrochaites* in 2019. In *S. corneliomulleri* (2019), the fungal genus *Filobasidium* (*p* =  < 0.001) was significantly more abundant in dysbiotic leaf samples. In contrast to *S. habrochaites* (2019), *Davidiella* was frequently found in healthy samples of *S. corneliomulleri* (2019). Dysbiotic samples from *S. habrochaites* and *S. corneliomulleri* displayed similar trends for *Cryptococcus*, *Erythrobasidium*, and Tremellomycetes. Nevertheless, total read counts of Dothideomycetes were contradictory between 18S rRNA amplicons (see Supplementary Fig. 6H) and ITS2 amplicons (see Supplementary Fig. 6I). Since the taxonomic resolution of Dothideomycetes (c) and Tremellomycetes (c) reached class level, we collected all representative sequences of OTUs to perform a nucleotide blast search against the NCBI nt database to verify taxonomic assignments. We searched 161 OTUs from 18S rRNA amplicons and 5500 OTUs from ITS2 amplicons against NCBI nt database and revealed that Dothideomycetes in 18S rRNA amplicons are mainly represented by pathogenic fungi, like *Stagonosporopsis* spp. (84 OTUs), *Boeremia lycopersici* (23 OTUs), *Leptosphaeria maculans* (14 OTUs), *Phoma medicaginis* (6 OTUs), and *Alternaria* spp. (3 OTUs). In addition, we found fungal pathogens known from other host plants, like *Parastagonospora nodorum* (14 OTUs) a pathogen in wheat and *Cercospora sojina* (11 OTUs) a pathogen in soybean. Further, 33 OTUs remained as uncultured or predicted microbes. In addition, we identified 5500 ITS2 OTUs, classified as Dothideomycetes. The majority, represented by 4307 OTUs, remained as uncultured microbes. Nevertheless, we were able to identify OTUs belonging to *Phoma* spp. (383 OTUs), *Cladosporium* spp. (253 OTUs), *Microsphaeropsis* spp. (197 OTUs), *Boeremia* spp (176 OTUs), and *Ascochyta* spp. (161 OTUs). Further fungal genera were classified as *Neomicrosphaeropsis* spp. (32 OTUs), *Xenodidymella* spp. (28 OTUs), *Epicoccum nigrum* (14 OTUs), *Didymella* spp. (12 OTUs), *Neodidymelliopsis* spp. (9 OTUs), *Dothiorella* spp. (7 OTUs), *Coniothyrium aleuritis* (5 OTUs), *Leptosphaerulina arachidicola* (4 OTUs), and *Septoria* spp. (2 OTUs). The fungal class Tremellomycetes (unclassified) in ITS2 was represented by low abundant Otu0004361 and Otu0006293.

We hypothesized an enrichment of plant beneficial microbes in healthy samples, whereas plant pathogens are dominated in dysbiotic samples. Our results support that hypothesis primarily. However, we observed inconsistencies between sampling years of *S. habrochaites* in 2018 and 2019.

#### Microbe-Microbe Interactions in Healthy and Dysbiotic Leaves

**Fig. 6:**
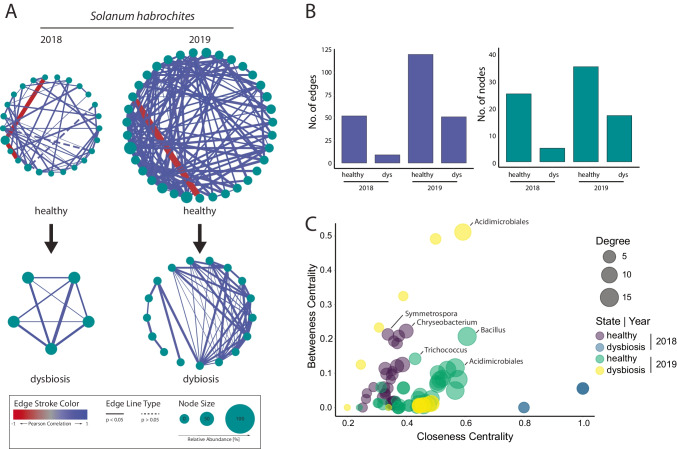
Changes of correlation networks of *S. habrochaites* between leaf symptoms in two consecutive sampling years.** A** Network analysis based on Pearson correlation coefficient ($\pm$ 0.6) on summarized taxa using Cytoscape (v.3.8.2). Degree sorted circular plots are shown for healthy and dysbiotic samples of *S. habrochaites* in two consecutive years. Edge stroke colors depict Pearson correlation coefficients between $\pm 1$. Edgle line type “unbroken line” shows significant differences (*p* < 0.05) between microbial taxa achieved by Wilcoxon-Tests (*p*-val. adjust: “Bonferroni”). Node size displays the relative abundance. **B** Number of edges and nodes of calculated correlation networks. **C** Identification of important microbial taxa within the phyllosphere microbiota. Circles are colored according to leaf symptoms (healthy, dysbiosis) and sampling years (2018, 2019). Circle sizes correspond to node degrees. Taxonomic labels indicate top 5% values of betweenness centrality and closeness centrality within each correlation network

Microbe-microbe interactions have been shown to impact microbial consortia on the phyllosphere and rhizosphere of various host plants. In this section, we aimed to identify cross-kingdom interaction in healthy and dysbiotic leaves of *S. habrochaites* on summarized taxa to generate correlation networks over 2 consecutive years. Summarized taxa were generated as described in the previous section. Pearson correlation matrices were calculated for each host species, sampling year, and leaf symptoms. A threshold for Pearson correlation coefficients was set to $\pm$ 0.6, to be considered in the calculation of co-occurrence networks (see Fig. 6A). Network characteristics like number of nodes and edges and degree, as well as betweenness centrality and closeness centrality, were used to compare main networks (see Fig. 6B–C). Thereby, calculated networks from healthy samples displayed a higher complexity in respect to the number of edges and nodes, in contrast to dysbiotic samples. Over all networks, most Pearson correlation coefficients are positively correlated. Nevertheless, in networks comprising healthy samples, we observed negative correlations between the fungal class of Cystobasidiomycetes and bacterial (*Patulibacter*) or fungal (*Slooffia*) genera in 2018. In 2019, the fungal class of Dothideomycetes was negatively correlated to the bacterial genera *Lectera* on the epiphytic phyllosphere.

Further, we calculated two main network centralities that indicate how nodes (summarized taxa) are connected to each other. Betweenness centrality measures the number of shortest paths between the node of interest and other connected nodes, which is used to quantify the centrality of a node in a network. In parallel, closeness centrality was calculated, which represents “the reciprocal of the sum of distances to all other nodes.” Those measurements were previously used by [2] to identify “hub” microbes (“significantly more connected nodes within the network then other nodes”). Our network analysis was performed on summarized taxa (concatenation of single OTUs belonging to the lowest taxonomic rank). To do so, the top 5% of summarized taxa showing the highest betweenness and closeness centrality with the main networks were considered hub microbial taxa (see Fig. 6C). Hub microbial taxa belonging to bacteria genera like *Bacillus* (g), *Chryseobacterium* (g), *Acidimicrobiales* (o), and *Trichococcus* (g) as well as ballistosporous yeasts of *Symmetrospora* (g) were identified for *S. habrochaites*. Interestingly, the majority of microbial hub taxa are common in healthy leaves, such as Bacillus (*p* = 0.0008). In contrast, *Chryseobacterium* were abundant in dysbiotic leaves in 2018, whereas low abundant in 2019. The bacterial order of *Acidimicrobiales* was detected as hub microbial taxon across *S. habrochaites* leaves. Curiously, *Acidimicrobiales* was more abundant in healthy leaves in 2019 (*p* = 0.0093). The bacterial genus *Trichococcus* belongs to rare taxa and was not considered for further conclusions. Yeast hub taxa belonging to *Symmetrospora* were identified and considered as highly abundant in dysbiotic leaves in 2019 (*p* = 0.0001).

Those findings from a multi-kingdom network analysis on summarized hub taxa support our hypothesis of higher frequencies of plant promoting microbial taxa in healthy leaves, represented by *Bacillus* (g) and *Acidimicrobiales* (o). On the other site, plant pathogenic microorganisms were highly abundant in dysbiotic leaves, displayed by the bacterial genus *Chryseobacterium* and yeast genus *Symmetrospora*.

## Discussion

### Phyllosphere Microbiome Impacted by Tomato Species Across Geographical Locations

Microbial consortia are important cofactors for plant health [100]. Yet, limited knowledge exists about microbiomes of wild plant species and crop wild relatives. We applied an amplicon-based sequencing approach on bacteria, eukaryotes, and fungi to obtain an overview of the microbial phyllosphere consortia in wild tomato species. Initially, we investigated how microbial richness and diversity varies across host species in two geographical locations (Host × Year). Our data suggests that bacterial and eukaryotes diversities are significantly affected by host species and geographical location. Notably, various studies supporting our findings on host genotype and geographical origin impact microbial community structures [64, 83, 98]. Epiphytic fungal richness remained stable across host species and geographical origin. A study of Xiong et al. [100] discovered that epiphytic fungal taxa are widespread across geographical locations. While abundant taxa of the endophytic mycobiome are more stable across environmental conditions, rare taxa are more sensitively shaped by host selections [100]. In detail, we observed more diverse bacterial composition in *S. pimpinellifolium*, compared to *S. corneliomulleri* and *S. habrochaites*. In contrast, eukaryotes displayed lower diversities in *S. pimpinellifolium* compared to *S. habrochaites*. Phenotypically, plant architectures, such as leaf shape, lead branching, leaf area, and plant height, vary among wild tomatoes [65], which might affect our phyllosphere microbiota comparison in relation to microbes per leaf sample. Additionally, a multivariate analysis on Bray–Curtis dissimilarities identified the sampling year as a major driver of beta diversity in our dataset. We also observed a host species effect on the phyllosphere microbiome, which was strongly supported by *S. habrochaites* and *S. corneliomulleri* present in Canta sites. In contrast, beta diversities from *S. peruvianum* and *S. pimpinellifolium* displayed a high overlap, which could be explained by the smaller distances between the populations around Yangas.

By comparing microbial compositions between host and year, we identified Proteobacteria, Actinobacteria, Firmicutes, and Bacteroides as most prominent bacterial phyla on tomato leaf surfaces. Notably, such bacterial phyla dominate bacterial communities in various plant species [21, 33, 38]. Interestingly, relative abundances of Proteobacteria were higher in Canta, whereas Firmicutes were more abundant in Yangas. A recent study of T. Chen et al. [22] on *Arabidopsis thaliana* evaluated a counteractive correlation of endophytic Proteobacteria and Firmicutes in PTI mutant lines. The relative abundance of epiphytic Proteobacteria and Firmicutes were altered by powdery mildew on *E. japonicus* [105]. Such observations raise questions about the role of phyllosphere microbiota in regulating PTI in wild tomato species, as large quantitative variations can be seen in resistance properties and PTI responses within single wild tomato species (Stam, Scheikl, and Tellier 2017,[53].

Eukaryotic microbial communities of wild tomatoes were mainly composed of Opisthokonta, representing a broad range of eukaryotes, such as fungi and protists. Thereby, protists have been identified as key determinants for plant performance [44]. The phylum Stramenopiles, including the foliar oomycete *Phytophthora infestans* (causal agent of late blight disease in tomato), was rarely detectable in Canta and low abundant in Yangas. Samplings of wild tomato leaves were conducted during dry seasons. Thus, low abundances of *Pinf* might be related to the oomycete life cycle, since hot and dry conditions are not appropriate for sporulation [77]. On the other side, multiple likely resistance gene loci against *Pinf* have been identified in wild tomato species underlying local adaptation [84, 85,86,87]. Fungal taxa in wild tomatoes were dominated by Ascomycota and Basidiomycota, representing the most abundant phyla on plants [11, 41].

Since multiple factors were determined to impact microbial community compositions, we aimed to identify consistent microbes across the whole dataset. Thereby, *Pseudomonas*, *Methylobacterium*, and *Sphingomonas* are the most abundant bacterial genera. *Pseudomonas* has been widely described in the phyllosphere across host species [33]. Hence, plant beneficial and pathogenic species have been identified. It has been shown that facultative methylotrophic bacteria of the genus *Methylobacterium* are able to promote tomato growth, biomass and fruit yield [50, 80]. Bacterial genera like *Pseudomonas*, *Sphingomonas*, *Bacillus*, and *Methylobacterium* have been described as microbial core members on the phyllosphere [43, 74]. Several studies indicate that single strains of *Sphingomonas* and *Microbacterium* show the highest potential to affect microbial community structures [16]. *Methylobacterium adhaesivum* has been described as a novel species in a drinking water distribution system of Spain [40]. However, nothing is known about the relevance in microbial phyllosphere communities to our knowledge. In addition, *Variovorax* was found as a microbial core member. *Variovorax* was identified as a key player of the root microbiome, affecting auxin degradation processes in Arabidopsis and tomato [31]. On the phyllosphere, *Variovorax* is involved in degradation of isoprene carbon sources, which might be produced by the plant under stress conditions [28, 35, 51].

Eukaryotic core microbes, comprising fungi, yeasts, and eukaryotes, were represented by *Davidiella tassiana*, *Phoma medicaginis*, *Cryptococcus*, *Vishniacozyma*, *Alternaria*, *Erythrobasidium*, and Tremellomycetes. In addition, we obtained core microbes by 18S rRNA amplicons belonging to *Ophistosphaerella*, *Aureobasidium pullulans*, *Symmetrospora*, and Dothideomycetes. Notably, fungal classes like Tremellomycetes and Dothideomycetes have been identified as fungal core taxa in Anthurium spp. [5]. Interestingly, *Phoma medicaginis*, the causal agent of spring black stem of *Medicago truncatula*, was identified as a core member of the tomato. However, the polyphyletic genus *Phoma* contains more than 3000 species, including important plant pathogens as well as plant protecting species [30]. The endophytic *Phoma eupatorii* has been described as a biocontrol agent, which conveys broad-spectrum inhibition of *Pinf* on tomato [97]. The saprophytic yeast-like fungus *Aureobasidium pullulans* shows antimicrobial properties, which makes them suitable as bioagents for bacteria and fungi pathogens [72].

Fungal core microbes were identified as *Cladosporium*, *Filobasidium*, *Symmetrospora*, and *Erythrobasidium hasegawianum*. The fungal genus Cladosporium includes commonly leaf mold pathogens (renamed to *Passalora fulva*) found epiphytic and endophytic on tomato and other host species [49, 89]. Multiple resistance genes have been described in wild tomatoes against *Cladosporium fulvum*. These genes are conserved between wild tomato species, but also show large within species allelic variation, suggesting an important role for the interaction of *C. fulvum* with wild tomato [52, 57, 96]. In our dataset, the fungal genera *Filobasidium*, *Symmetrospora*, and *Erythrobasidium*, belonging to basidiomycete yeasts, were identified as microbial core taxa on the tomato phyllosphere. To our knowledge, here we described the first survey of *Symmetrospora*, representing yeasts occurring on tomato leaf surfaces. These red basidiomycete yeasts, where previously phylogenetically described as *Sporobolomycetes* and have been found on tobacco [45, 104]. Their role in microbial communities remains unknown. However, basidiomycete yeast fungi, like *Dioszegia*, have been described as hub microbes on the phyllosphere of Arabidopsis [2].

Our results suggest various factors imparting epiphytic microbial communities on wild tomato species, such as sampling year, geographical origin, and host-specific selection. We observed a host species effect on diversities of microbial communities, mainly visible in bacteria and protists. In terms of diversity, fungal community compositions remained rather stable across mentioned data features. Independently of host species, we observed persistent core microbes for bacteria, yeasts, and fungi.

### Compositional Changes of Microbes upon Dysbiosis in Canta

Microbiome studies revealed that pathogenic microbes highly impact microbial community structures [2]. In addition to microbiome communities, the proliferation of plant pathogens is influenced by microbial environmental conditions and host resistotypes [12, 23, 55]. Due to the availability of large numbers of samples, we could perform a downstream analysis implementing leaf symptoms for microbiome samples from wild tomatoes in Canta. By grouping healthy and dysbiotic samples according to host species, sampling year, and leaf symptoms (Host × Year × Symptom), we obtained affected microbial richness between groups. Richness of bacteria and eukaryotes were affected. Healthy samples taken in 2019 showed higher bacterial richness, compared to dysbiotic leaves. Interestingly, Firmicutes were less abundant in dysbiotic samples of *S. habrochaites* (2019), compared to higher abundances of Proteobacteria. Notably, disruption of Firmicutes have been linked to dysbiosis in the phyllosphere and rhizosphere [22, 62]. Eukaryotic richness was lower in healthy samples compared to dysbiotic leaves. Further, fungal richness remained stable across Host × Year × Symptom, which indicates robust fungal community structures. In contrast, *S. habrochaites* sampled in 2018 showed comparable richness of bacteria and eukaryotes. This lack of differences can potentially be attributed to the fact that in 2018, the Canta samples were collected under rainy conditions. Splashes and runoff could have affected microbial composition of healthy and dysbiotic leaves in either direction. Further, we observed interspecies differences between dysbiotic leaves of *S. corneliomulleri*, which was significantly lower compared to *S. habrochaites* in 2 consecutive years. Conclusively, microbial richness tends to be lower for bacteria and higher for eukaryotes in dysbiotic tomato leaves. Fungal richness remained stable across Host × Year × Symptom.

Since microbial richness was affected by host species and sampling years, we conducted a multi-variance analysis using Bray–Curtis dissimilarities to display beta diversities in respect to leaf symptoms. Since beta diversities cluster distinctly between healthy and dysbiotic samples collected in Canta, we calculated Pearson correlation networks for summarized taxa of *S. habrochaites* of 2 consecutive years respectively. Network characteristics, such as number of edges and nodes, degree, and network centralities, were used to identify microbial hub taxa in relation to leaf symptoms. Hub microbes have been suggested as important microbiome members affecting community stability and structure [2, 7, 47]. We identified microbial hub taxa in healthy samples of *S. habrochaites*, such as *Chryseobacterium*, *Symmetrospora* (2018), *Bacillus*, and *Trichococcus* (2019). Natural *Bacillus* spp. (belonging to Firmicutes) have been widely described as a biocontrol agent against various plant pathogens, such as *Botrytis cinerea* or *Cladosporium fulvum*, *Fusarium oxysporum*, *Pythium aphanidermatum*, *Colletotrichum capsici*, and *Sclerotium rolfsii* [4, 18, 54, 99]. The class of Acidimicrobiales was detected as hub taxa in healthy and dysbiotic samples from 2019, which might play a major role in microbial phyllosphere communities of tomato independent of dysbiosis,Acidimicrobiales have previously been identified as a keystone species of tomato rhizosphere microbiota [62]. The Actinobacteria Acidimicrobiales have been dominantly found in nutrient-rich soil [73]. Interestingly, further hub taxa could not be detected in dysbiotic samples, indicating that leaves in dysbiosis can be host to a much large variety of taxa than healthy samples. Rare fungal taxa have been identified as hub microbes on maize, affecting microbial community stabilities [100]. Conclusively, our data suggest numerous factors influencing phyllosphere microbiota on wild tomato species. While sampling year and geographical origin are favored factors shaping the phyllosphere microbiome, host genotypes of wild tomatoes affected microbial community assemblies. Further, dysbiotic leaves of *S. habrochaites* and *S. corneliomulleri* displayed clear altered microbial composition and multiple hub taxa related to plant health. These data form a robust starting point for follow-up studies aimed at understanding the role of the phyllosphere microbiome composition in pathogen resistance in natural plant populations of crop wild relatives.

## Supplementary Information

Below is the link to the electronic supplementary material.

Supplementary file1 (PDF 1547 KB)

## Data Availability

Supporting data and material of the current study are available within the paper and it’s Supplementary Information section. The generated and analyzed dataset of this study are available from the corresponding author upon request. Raw sequencing data is accessible through the European Nucleotide Archive under the corresponding project accession number PRJEB45304. An overview of submitted sequencing data and sample information are summarized in Supplementary Table 2. The source data of Figs.1–6 and Supplementary Figs. 1–6 are provided as a source data file.

## Code Availability

Custom scripts used in this study are freely accessible at https://github.com/RUNGEP/TomPhylloMicro.
