# Supplementary material for: Distinct Phyllosphere Microbiome of Wild Tomato Species in Central Peru upon Dysbiosis
Source: Microb Ecol. 2022 Jan 18;85(1):168–83. doi: 10.1007/s00248-021-01947-w (PMC9849306; doi:10.1007/s00248-021-01947-w)
Supplement: Supplementary file 1 — Supplementary file1 (PDF 1547 KB) [file 248_2021_1947_MOESM1_ESM.pdf]

Supplementary Information for:

Distinct phyllosphere microbiome of wild tomato species in central Peru upon dysbiosis

Authors: Paul Runge[#1,2], Freddy Ventura[3], Eric Kemen[#1], Remco Stam[*4]

[1] Department of Microbial Interactions, IMIT/ZMBP, University of Tübingen, Tübingen, Germany

[2] Department of Plant Microbe Interactions, Max Planck Institute for Plant Breeding Research, Carl-von-Linne-Weg 10, 50829 Köln, Germany.

[3] Plant Pathology and Bacteriology, International Potato Centre, Avenida La Molina 1895, La Molina, Lima, Peru

[4] Chair of Phytopathology, TUM School of Life Science, Emil-Ramann-Str. 2, 85354 Freising-Weihenstephan, Germany

* correspondence to: Remco Stam, remco.stam@tum.de

ORCID: 0000-0001-8927-8524, 0000-0002-7924-116X, 0000-0002-3444-6954

- Abstract -

Plants are colonized by myriads of microbes across kingdoms, which affect host development, fitness and reproduction. Hence, plant microbiomes have been explored across a broad range of host species, including model organisms, crops and trees under controlled and natural conditions. Tomato is one of the world's most important vegetable crops, however little is known about the microbiota of wild tomato species. To obtain insights into the tomato microbiota occurring in natural environments, we sampled epiphytic microbes from leaves of four tomato species, *Solanum habrochaites*, *S. corneliomulleri*, *S. peruvianum* and *S. pimpinellifolium,* from two geographical locations within the Lima region of Peru over two consecutive years. Here, a high-throughput sequencing approach was applied to investigate microbial compositions including bacteria, fungi and eukaryotes across tomato species and geographical locations. The phyllosphere microbiome composition varies between hosts and location. Yet, we identified persistent microbes across tomato species that form the tomato microbial core community. In addition, we phenotypically defined healthy and dysbiotic samples and performed a downstream analysis to reveal the impact on microbial community structures. To do so, we compared microbial diversities, unique OTUs, relative abundances of core taxa and microbial hub taxa, as well as co-occurrence network characteristics in healthy and dysbiotic tomato leaves and found that dysbiosis affects the phyllosphere microbial composition in a host species-dependent manner Yet, overall, the present data suggests an enrichment of plant-promoting microbial taxa in healthy leaves, whereas numerous microbial taxa containing plant pathogens occurred in dysbiotic leaves.

# - Supplementary Information -

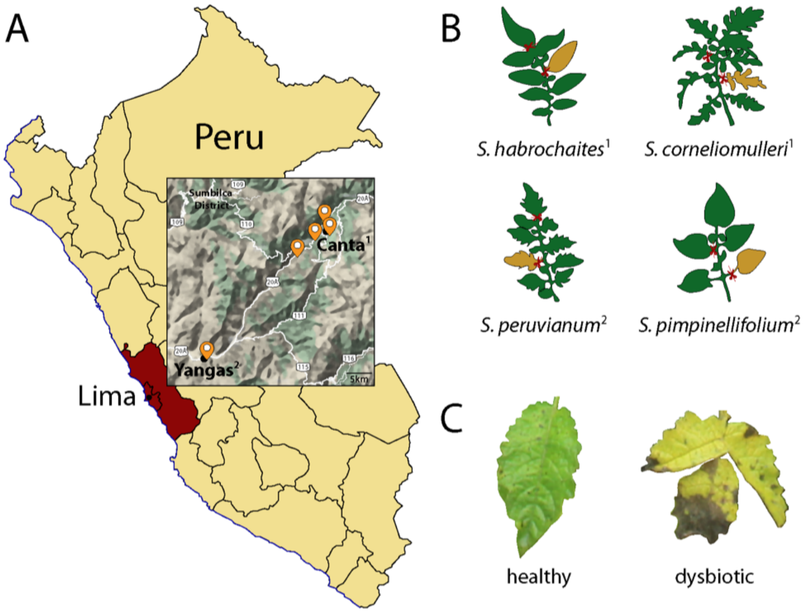

Supplementary Fig. 1: Characteristics of sampled wild tomato species within the Lima region of Peru. A) Map of Peru. Highlighted in red is the Lima province. The inset shows the specific sampling sites around Canta and Yangas. B) Visualization of the distinguishable leaf morphologies for *S. habrochaites* and *S. corneliomulleri* (Canta), *S. peruvianum* and *S. pimpinellifolium* (Yangas). C) Phenotypic classification of healthy and dysbiotic leaves exemplified with *S. habrochaites leaves*.

Microbial richness varies across geographical origin and years

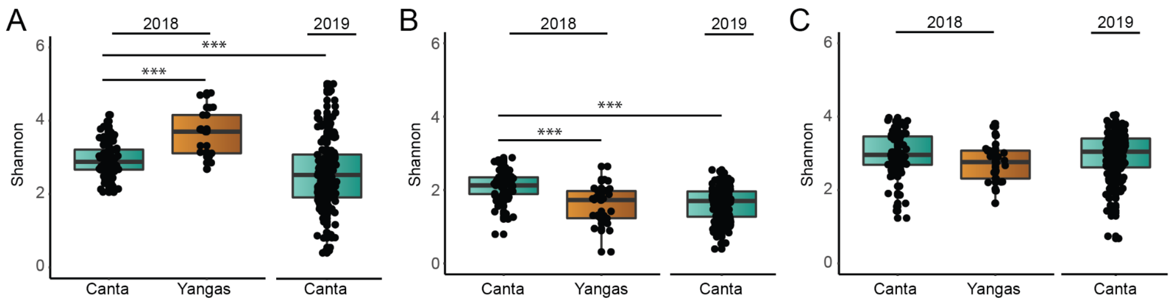

Supplementary Fig. 2: Microbial richness across geographical origin and sampling years. Shannon indices (alpha diversity) were calculated for bacteria (A), eukaryotes (B) and fungi (C). Pairwise-Wilcoxon-Test was performed with "Bonferroni" p-value adjustment using R, *** = <0.001.

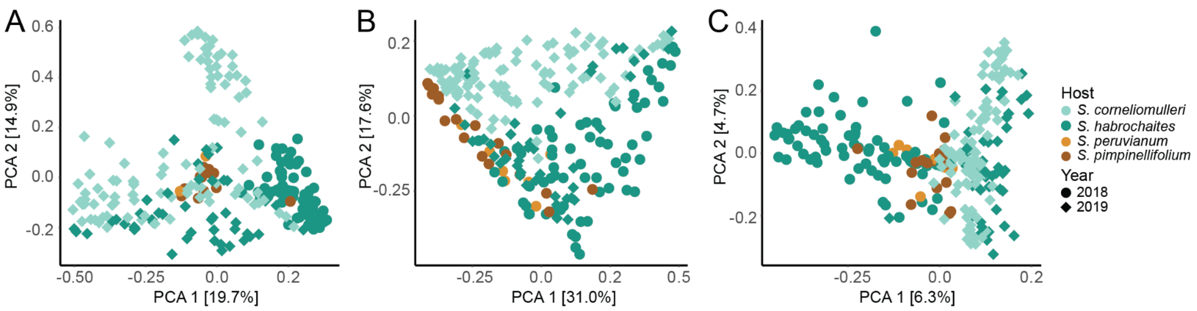

Supplementary Fig. 3: Principal component analysis (PCA) of microbiome samples in relation to tomato species and sampling years. PCA's were conducted for A) bacteria, B) eukaryotes and c) fungi. Shown is the PCA on Bray-Curtis dissimilarities displaying two dimensions PCA 1 and PCA2 in the ordination system. Each point represents one leaf sample colored by host species and shaped by sampling years. Permanova (P 999) was conducted using qiime2; $p < 0.001$.

Relative abundance of microbial profiles of *Solanum* spp. on phylum level

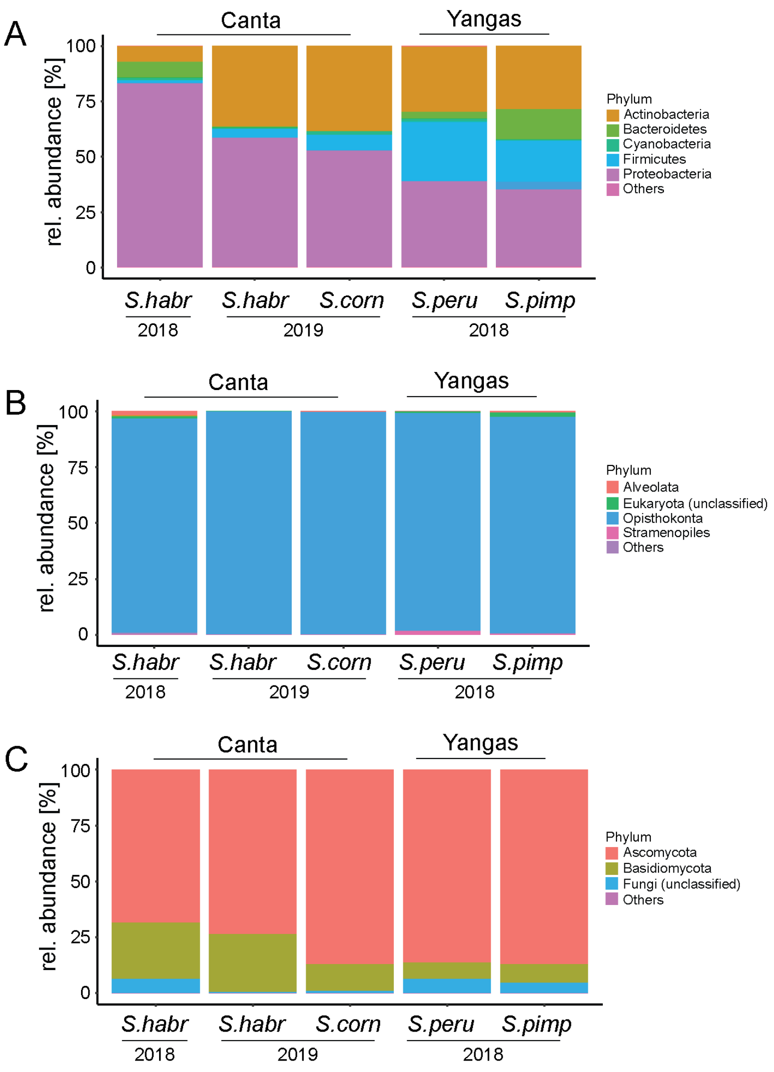

Supplementary Fig. 4: Relative abundance plots rendering host species and sampling years on phylum level. Abbreviations are related to *S. habrochaites* (*S. habr*), *S. cornelimulleri* (*S. corn), S. peruvianum* (*S. peru*) and *S. pimpinellifolium* (*S. pimi*). A) Bacterial 16S rRNA, B) eukaryotic 18S rRNA, C) fungal ITS2.

Supplementary Tab. 1: Pairwise Wilcoxon-Test (p.adjust: "bonferroni") comparing tomato species and sampling year against each phyla.

Amplicon	Phylum	Group1	Group 2	p-value
16S rRNA	Actinobacteria	*S. peruvianum* (2018)	*S. habrochaites* (2018)	8.05E-39
16S rRNA	Actinobacteria	*S. peruvianum* (2018)	*S. corneliomulleri* (2019)	7.82E-29
16S rRNA	Actinobacteria	*S. peruvianum* (2018)	*S. habrochaites* (2019)	2.58E-21
16S rRNA	Actinobacteria	*S. pimpinellifolium* (2018)	*S. habrochaites* (2018)	3.09E-20
16S rRNA	Actinobacteria	*S. habrochaites* (2019)	*S. habrochaites* (2018)	4.96E-11
16S rRNA	Actinobacteria	*S. pimpinellifolium* (2018)	*S. peruvianum* (2018)	3.70E-09
16S rRNA	Actinobacteria	*S. pimpinellifolium* (2018)	*S. corneliomulleri* (2019)	1.98E-08
16S rRNA	Actinobacteria	*S. corneliomulleri* (2019)	*S. habrochaites* (2018)	9.49E-05
16S rRNA	Actinobacteria	*S. pimpinellifolium* (2018)	*S. habrochaites* (2019)	9.47E-04
16S rRNA	Actinobacteria	*S. corneliomulleri* (2019)	*S. habrochaites* (2019)	2.26E-02
16S rRNA	Bacteroidetes	*S. pimpinellifolium* (2018)	*S. corneliomulleri* (2019)	6.84E-09
16S rRNA	Bacteroidetes	*S. peruvianum* (2018)	*S. corneliomulleri* (2019)	6.78E-07
16S rRNA	Bacteroidetes	*S. peruvianum* (2018)	*S. habrochaites* (2018)	1.81E-05
16S rRNA	Bacteroidetes	*S. peruvianum* (2018)	*S. habrochaites* (2019)	3.52E-05
16S rRNA	Bacteroidetes	*S. corneliomulleri* (2019)	*S. habrochaites* (2018)	6.75E-05
16S rRNA	Bacteroidetes	*S. pimpinellifolium* (2018)	*S. habrochaites* (2018)	1.09E-04
16S rRNA	Bacteroidetes	*S. pimpinellifolium* (2018)	*S. habrochaites* (2019)	2.40E-04
16S rRNA	Chloroflexi	*S. corneliomulleri* (2019)	*S. habrochaites* (2019)	7.78E-07
16S rRNA	Chloroflexi	*S. habrochaites* (2019)	*S. habrochaites* (2018)	1.47E-05
16S rRNA	Firmicutes	*S. peruvianum* (2018)	*S. habrochaites* (2018)	1.40E-36
16S rRNA	Firmicutes	*S. peruvianum* (2018)	*S. corneliomulleri* (2019)	3.09E-33
16S rRNA	Firmicutes	*S. pimpinellifolium* (2018)	*S. habrochaites* (2018)	2.84E-31
16S rRNA	Firmicutes	*S. peruvianum* (2018)	*S. habrochaites* (2019)	1.31E-30
16S rRNA	Firmicutes	*S. pimpinellifolium* (2018)	*S. corneliomulleri* (2019)	3.09E-22
16S rRNA	Firmicutes	*S. pimpinellifolium* (2018)	*S. habrochaites* (2019)	4.08E-19
16S rRNA	Firmicutes	*S. pimpinellifolium* (2018)	*S. peruvianum* (2018)	1.60E-08
16S rRNA	Firmicutes	*S. habrochaites* (2019)	*S. habrochaites* (2018)	3.36E-06
16S rRNA	Firmicutes	*S. corneliomulleri* (2019)	*S. habrochaites* (2018)	1.33E-04
16S rRNA	Fusobacteria	*S. pimpinellifolium* (2018)	*S. habrochaites* (2018)	6.48E-03
16S rRNA	Proteobacteria	*S. peruvianum* (2018)	*S. corneliomulleri* (2019)	2.35E-45
16S rRNA	Proteobacteria	*S. peruvianum* (2018)	*S. habrochaites* (2018)	2.86E-38
16S rRNA	Proteobacteria	*S. peruvianum* (2018)	*S. habrochaites* (2019)	8.39E-37
16S rRNA	Proteobacteria	*S. pimpinellifolium* (2018)	*S. corneliomulleri* (2019)	8.88E-20
16S rRNA	Proteobacteria	*S. pimpinellifolium* (2018)	*S. peruvianum* (2018)	1.02E-17
16S rRNA	Proteobacteria	*S. pimpinellifolium* (2018)	*S. habrochaites* (2019)	9.21E-09
16S rRNA	Proteobacteria	*S. corneliomulleri* (2019)	*S. habrochaites* (2018)	1.05E-07
16S rRNA	Proteobacteria	*S. pimpinellifolium* (2018)	*S. habrochaites* (2018)	1.85E-06
16S rRNA	Proteobacteria	*S. corneliomulleri* (2019)	*S. habrochaites* (2019)	1.30E-05

18S rRNA	Amoebozoa	*S. pimpinellifolium* (2018)	*S. habrochaites* (2018)	3.20E-03
18S rRNA	Eukaryota_unclassified	*S. peruvianum* (2018)	*S. habrochaites* (2019)	1.83E-03
18S rRNA	Eukaryota_unclassified	*S. peruvianum* (2018)	*S. corneliomulleri* (2019)	1.86E-03
18S rRNA	Eukaryota_unclassified	*S. habrochaites* (2019)	*S. habrochaites* (2018)	5.63E-03
18S rRNA	Eukaryota_unclassified	*S. corneliomulleri* (2019)	*S. habrochaites* (2018)	1.16E-02
18S rRNA	Eukaryota_unclassified	*S. pimpinellifolium* (2018)	*S. habrochaites* (2019)	2.76E-02
18S rRNA	Opisthokonta	*S. peruvianum* (2018)	*S. corneliomulleri* (2019)	5.08E-21
18S rRNA	Opisthokonta	*S. peruvianum* (2018)	*S. habrochaites* (2019)	2.14E-16
18S rRNA	Opisthokonta	*S. peruvianum* (2018)	*S. habrochaites* (2018)	1.14E-13
18S rRNA	Opisthokonta	*S. pimpinellifolium* (2018)	*S. corneliomulleri* (2019)	3.79E-12
18S rRNA	Opisthokonta	*S. pimpinellifolium* (2018)	*S. habrochaites* (2019)	1.84E-07
18S rRNA	Opisthokonta	*S. corneliomulleri* (2019)	*S. habrochaites* (2018)	7.20E-06
18S rRNA	Opisthokonta	*S. pimpinellifolium* (2018)	*S. peruvianum* (2018)	6.67E-05
18S rRNA	Opisthokonta	*S. pimpinellifolium* (2018)	*S. habrochaites* (2018)	1.15E-03
ITS2	Ascomycota	*S. peruvianum* (2018)	*S. corneliomulleri* (2019)	1.21E-245
ITS2	Ascomycota	*S. pimpinellifolium* (2018)	*S. corneliomulleri* (2019)	1.19E-227
ITS2	Ascomycota	*S. peruvianum* (2018)	*S. habrochaites* (2018)	2.61E-210
ITS2	Ascomycota	*S. pimpinellifolium* (2018)	*S. habrochaites* (2018)	4.78E-159
ITS2	Ascomycota	*S. corneliomulleri* (2019)	*S. habrochaites* (2019)	3.70E-151
ITS2	Ascomycota	*S. peruvianum* (2018)	*S. habrochaites* (2019)	1.43E-120
ITS2	Ascomycota	*S. habrochaites* (2019)	*S. habrochaites* (2018)	5.84E-78
ITS2	Ascomycota	*S. pimpinellifolium* (2018)	*S. habrochaites* (2019)	7.64E-55
ITS2	Ascomycota	*S. pimpinellifolium* (2018)	*S. peruvianum* (2018)	1.04E-24
ITS2	Ascomycota	*S. corneliomulleri* (2019)	*S. habrochaites* (2018)	1.00E-16
ITS2	Basidiomycota	*S. peruvianum* (2018)	*S. corneliomulleri* (2019)	6.03E-14
ITS2	Basidiomycota	*S. peruvianum* (2018)	*S. habrochaites* (2018)	3.30E-12
ITS2	Basidiomycota	*S. corneliomulleri* (2019)	*S. habrochaites* (2019)	1.28E-10
ITS2	Basidiomycota	*S. pimpinellifolium* (2018)	*S. corneliomulleri* (2019)	3.05E-10
ITS2	Basidiomycota	*S. habrochaites* (2019)	*S. habrochaites* (2018)	2.14E-08
ITS2	Basidiomycota	*S. pimpinellifolium* (2018)	*S. habrochaites* (2018)	1.38E-07
ITS2	Basidiomycota	*S. peruvianum* (2018)	*S. habrochaites* (2019)	2.22E-05
ITS2	Fungi_unclassified	*S. pimpinellifolium* (2018)	*S. habrochaites* (2019)	3.23E-02

Beta-dispersion analysis on Bray-Curtis dissimilarities to compare sample-to-sample variation

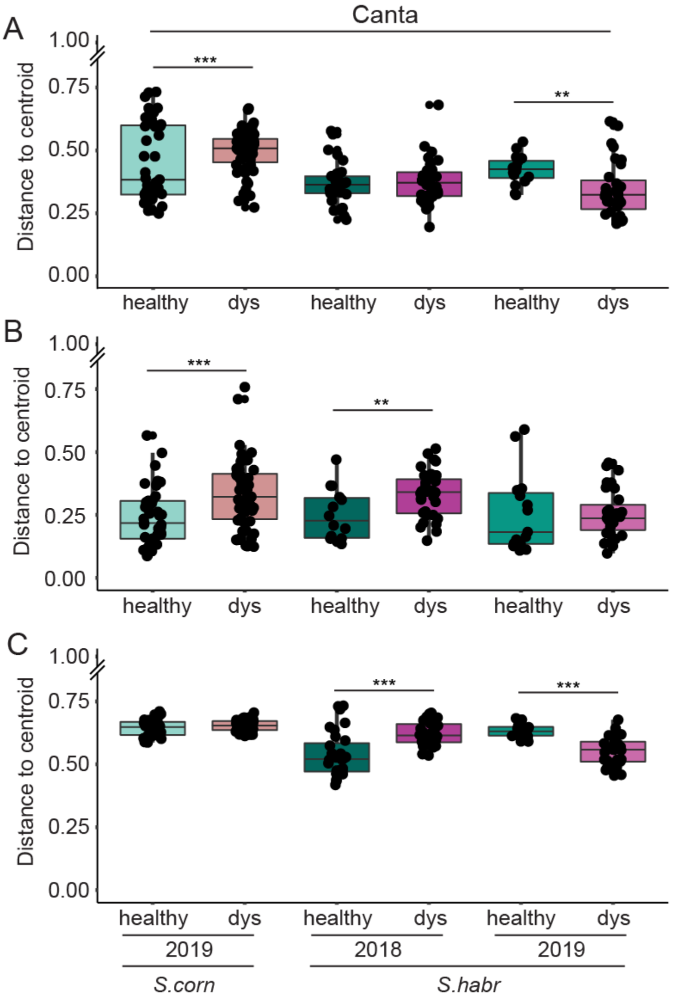

Supplementary Fig. 5: Beta-dispersion analysis on Bray-Curtis dissimilarities to compare sample-to-sample variation. Distance to centroids ("median") are displayed for data features Host x Year x Symptom in A) bacteria, B) eukaryotes and C) fungi. Statistics: Dunn test, ** $p < 0.01$, *** $p < 0.001$.

Shared and unique OTUs in tomato species across Canta in healthy and dysbiotic samples

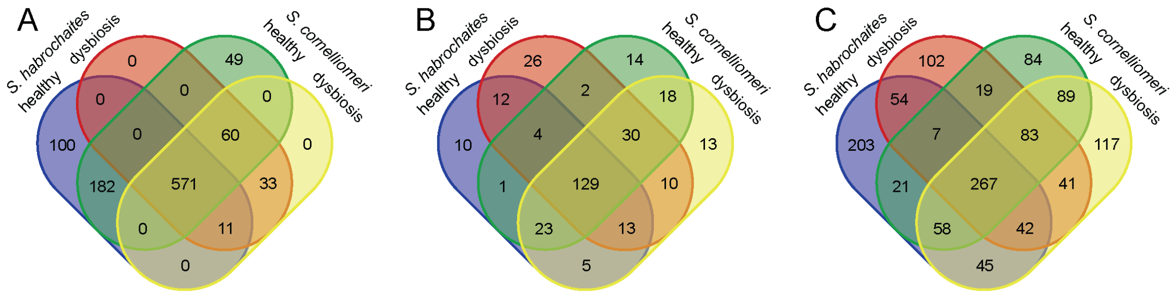

Supplementary Fig. 6: Shared and unique OTUs in Canta tomato species in healthy and dysbiotic samples. Venn-Diagrams showing occurrence of OTUs (A: bacteria, B: eukaryotes, C: fungi) within and between *S. habrochaites* and *S. corneliomulleri* in healthy

Total read counts of summarized core taxa in healthy and dysbiotic leaves

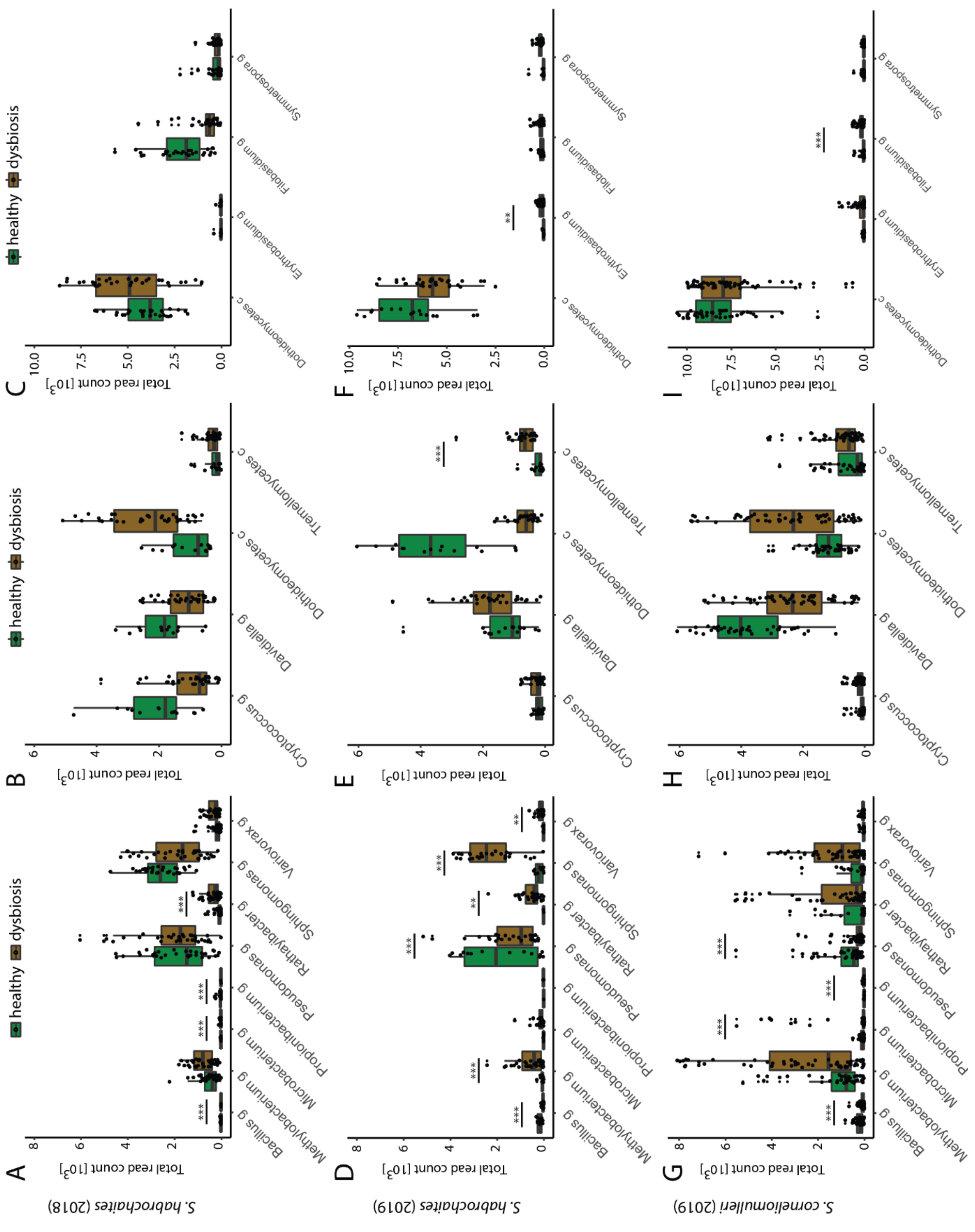

Supplementary Fig. 7: Total read counts of summarized core taxa on genus (g) or class (c) level across tomato species upon dysbiosis in Canta. ABC) *S. habrochaites* 2018. DEF) *S. habrochaites* 2019. GHI) *S. corneliomulleri* 2019. Pairwise-Wilcoxon-Test between healthy and dysbiotic samples was performed with "Bonferroni" p-value adjustment using R, *** = <0.001 ; ** <0.01, * <0.05.

ENA sample submission information

Supplementary Tab. 2: Sample metadata information paste to the European Nucleotide Archive containing sample collection data and ENA submission details.

Host Species	Year	Location	Area	Latitude	Longitude	Site	Plant	State	Bacteria [16S V5]		Eukaryotes [18S V9]		Fungi [ITS2]	
									Library	ENA Accession	Library	ENA Accession	Library	ENA Accession
S. peruvianum	2018	Peru	Yangas	-11.69946	-76.84604	Site10B	15	infected	CD.B5_1	ERS6577294(SAMEA8894384)	CD.P9s_1	ERS6577554(SAMEA8894644)	D.F2_1	ERS6577814(SAMEA8894905)
S. habrochaites	2018	Peru	Canta	-11.5130915	-76.678468	Site54	54	healthy	CD.B5_2	ERS6577295(SAMEA8894385)	CD.P9s_2	ERS6577555(SAMEA8894645)	D.F2_2	ERS6577815(SAMEA8894906)
S. habrochaites	2018	Peru	Canta	-11.5130915	-76.678468	Site54	56	healthy	CD.B5_3	ERS6577296(SAMEA8894386)	CD.P9s_3	ERS6577556(SAMEA8894646)	D.F2_3	ERS6577816(SAMEA8894907)
S. habrochaites	2018	Peru	Canta	-11.4806049	-76.6372699	Site52	41	healthy	CD.B5_4	ERS6577297(SAMEA8894387)	CD.P9s_4	ERS6577557(SAMEA8894647)	D.F2_4	ERS6577817(SAMEA8894908)
S. habrochaites	2018	Peru	Canta	-11.4806049	-76.6372699	Site52	42	infected	CD.B5_5	ERS6577298(SAMEA8894388)	CD.P9s_5	ERS6577558(SAMEA8894648)	D.F2_5	ERS6577818(SAMEA8894909)
S. habrochaites	2018	Peru	Canta	-11.5130915	-76.678468	Site53	45	healthy	CD.B5_6	ERS6577299(SAMEA8894389)	CD.P9s_6	ERS6577559(SAMEA8894649)	D.F2_6	ERS6577819(SAMEA8894910)
S. habrochaites	2018	Peru	Canta	-11.5130915	-76.678468	Site54	62	healthy	CD.B5_7	ERS6577300(SAMEA8894390)	CD.P9s_7	ERS6577560(SAMEA8894650)	D.F2_7	ERS6577820(SAMEA8894911)
S. habrochaites	2018	Peru	Canta	-11.5130915	-76.678468	Site54	49	infected	CD.B5_8	ERS6577301(SAMEA8894391)	CD.P9s_8	ERS6577561(SAMEA8894651)	D.F2_8	ERS6577821(SAMEA8894912)
S. habrochaites	2018	Peru	Canta	-11.5130915	-76.678468	Site53	46	healthy	CD.B5_9	ERS6577302(SAMEA8894392)	CD.P9s_9	ERS6577562(SAMEA8894652)	D.F2_9	ERS6577822(SAMEA8894913)
S. habrochaites	2018	Peru	Canta	-11.5130915	-76.678468	Site54	50	infected	CD.B5_10	ERS6577303(SAMEA8894393)	CD.P9s_10	ERS6577563(SAMEA8894653)	D.F2_10	ERS6577823(SAMEA8894914)
S. habrochaites	2018	Peru	Canta	-11.4806049	-76.6372699	Site52	39	infected	CD.B5_11	ERS6577304(SAMEA8894394)	CD.P9s_11	ERS6577564(SAMEA8894654)	D.F2_11	ERS6577824(SAMEA8894915)
S. pimpinellifolium	2018	Peru	Yangas	-11.69946	-76.84604	Site10B	18	healthy	CD.B5_12	ERS6577305(SAMEA8894395)	CD.P9s_12	ERS6577565(SAMEA8894655)	D.F2_12	ERS6577825(SAMEA8894916)
S. habrochaites	2018	Peru	Canta	-11.5130915	-76.678468	Site54	61	healthy	CD.B5_13	ERS6577306(SAMEA8894396)	CD.P9s_13	ERS6577566(SAMEA8894656)	D.F2_13	ERS6577826(SAMEA8894917)
S. habrochaites	2018	Peru	Canta	-11.5130915	-76.678468	Site54	54	infected	CD.B5_14	ERS6577307(SAMEA8894397)	CD.P9s_14	ERS6577567(SAMEA8894657)	D.F2_14	ERS6577827(SAMEA8894918)
S. habrochaites	2018	Peru	Canta	-11.4806049	-76.6372699	Site52	41	infected	CD.B5_15	ERS6577308(SAMEA8894398)	CD.P9s_15	ERS6577568(SAMEA8894658)	D.F2_15	ERS6577828(SAMEA8894919)
S. habrochaites	2018	Peru	Canta	-11.4806049	-76.6372699	Site52	40	healthy	CD.B5_16	ERS6577309(SAMEA8894399)	CD.P9s_16	ERS6577569(SAMEA8894659)	D.F2_16	ERS6577829(SAMEA8894920)
S. habrochaites	2018	Peru	Canta	-11.4806049	-76.6372699	Site52	41	infected	CD.B5_17	ERS6577310(SAMEA8894400)	CD.P9s_17	ERS6577570(SAMEA8894660)	D.F2_17	ERS6577830(SAMEA8894921)
S. habrochaites	2018	Peru	Canta	-11.5130915	-76.678468	Site54	55	healthy	CD.B5_18	ERS6577311(SAMEA8894401)	CD.P9s_18	ERS6577571(SAMEA8894661)	D.F2_18	ERS6577831(SAMEA8894922)
S. habrochaites	2018	Peru	Canta	-11.5130915	-76.678468	Site54	51	healthy	CD.B5_19	ERS6577312(SAMEA8894402)	CD.P9s_19	ERS6577572(SAMEA8894662)	D.F2_19	ERS6577832(SAMEA8894923)
S. habrochaites	2018	Peru	Canta	-11.5130915	-76.678468	Site53	43	infected	CD.B5_20	ERS6577313(SAMEA8894403)	CD.P9s_20	ERS6577573(SAMEA8894663)	D.F2_20	ERS6577833(SAMEA8894924)
S. habrochaites	2018	Peru	Canta	-11.5130915	-76.678468	Site54	55	healthy	CD.B5_21	ERS6577314(SAMEA8894404)	CD.P9s_21	ERS6577574(SAMEA8894664)	D.F2_21	ERS6577834(SAMEA8894925)
S. habrochaites	2018	Peru	Canta	-11.5130915	-76.678468	Site54	52	infected	CD.B5_22	ERS6577315(SAMEA8894405)	CD.P9s_22	ERS6577575(SAMEA8894665)	D.F2_22	ERS6577835(SAMEA8894926)
S. pimpinellifolium	2018	Peru	Yangas	-11.69946	-76.84604	Site10B	20	infected	CD.B5_23	ERS6577316(SAMEA8894406)	CD.P9s_23	ERS6577576(SAMEA8894666)	D.F2_23	ERS6577836(SAMEA8894927)
S. habrochaites	2018	Peru	Canta	-11.5130915	-76.678468	Site54	61	healthy	CD.B5_24	ERS6577317(SAMEA8894407)	CD.P9s_24	ERS6577577(SAMEA8894667)	D.F2_24	ERS6577837(SAMEA8894928)
S. habrochaites	2018	Peru	Canta	-11.4806049	-76.6372699	Site52	38	infected	CD.B5_25	ERS6577318(SAMEA8894408)	CD.P9s_25	ERS6577578(SAMEA8894669)	D.F2_25	ERS6577838(SAMEA8894929)
S. habrochaites	2018	Peru	Canta	-11.4806049	-76.6372699	Site52	37	healthy	CD.B5_26	ERS6577319(SAMEA8894409)	CD.P9s_26	ERS6577579(SAMEA8894670)	D.F2_26	ERS6577839(SAMEA8894930)
S. habrochaites	2018	Peru	Canta	-11.5130915	-76.678468	Site53	45	infected	CD.B5_27	ERS6577320(SAMEA8894410)	CD.P9s_27	ERS6577580(SAMEA8894671)	D.F2_27	ERS6577840(SAMEA8894931)
S. habrochaites	2018	Peru	Canta	-11.4806049	-76.6372699	Site52	36	infected	CD.B5_28	ERS6577321(SAMEA8894411)	CD.P9s_28	ERS6577581(SAMEA8894672)	D.F2_28	ERS6577841(SAMEA8894932)
S. habrochaites	2018	Peru	Canta	-11.5130915	-76.678468	Site54	53	infected	CD.B5_29	ERS6577322(SAMEA8894412)	CD.P9s_29	ERS6577582(SAMEA8894673)	D.F2_29	ERS6577842(SAMEA8894933)
S. habrochaites	2018	Peru	Canta	-11.5130915	-76.678468	Site54	56	healthy	CD.B5_30	ERS6577323(SAMEA8894413)	CD.P9s_30	ERS6577583(SAMEA8894674)	D.F2_30	ERS6577843(SAMEA8894934)
S. peruvianum	2018	Peru	Yangas	-11.69946	-76.84604	Site10B	17	infected	CD.B5_31	ERS6577324(SAMEA8894414)	CD.P9s_31	ERS6577584(SAMEA8894675)	D.F2_31	ERS6577844(SAMEA8894935)
S. habrochaites	2018	Peru	Canta	-11.5130915	-76.678468	Site53	43	infected	CD.B5_32	ERS6577325(SAMEA8894415)	CD.P9s_32	ERS6577585(SAMEA8894676)	D.F2_32	ERS6577845(SAMEA8894936)
S. habrochaites	2018	Peru	Canta	-11.4806049	-76.6372699	Site52	38	infected	CD.B5_33	ERS6577326(SAMEA8894416)	CD.P9s_33	ERS6577586(SAMEA8894677)	D.F2_33	ERS6577846(SAMEA8894937)
S. habrochaites	2018	Peru	Canta	-11.5130915	-76.678468	Site54	57	healthy	CD.B5_34	ERS6577327(SAMEA8894417)	CD.P9s_34	ERS6577587(SAMEA8894678)	D.F2_34	ERS6577847(SAMEA8894938)
S. habrochaites	2018	Peru	Canta	-11.5130915	-76.678468	Site54	60	healthy	CD.B5_35	ERS6577328(SAMEA8894418)	CD.P9s_35	ERS6577588(SAMEA8894679)	D.F2_35	ERS6577848(SAMEA8894939)
S. habrochaites	2018	Peru	Canta	-11.5130915	-76.678468	Site54	57	healthy	CD.B5_36	ERS6577329(SAMEA8894419)	CD.P9s_36	ERS6577589(SAMEA8894680)	D.F2_36	ERS6577849(SAMEA8894940)
S. habrochaites	2018	Peru	Canta	-11.5130915	-76.678468	Site54	53	healthy	CD.B5_37	ERS6577330(SAMEA8894420)	CD.P9s_37	ERS6577590(SAMEA8894681)	D.F2_37	ERS6577850(SAMEA8894941)
S. habrochaites	2018	Peru	Canta	-11.4806049	-76.6372699	Site52	36	healthy	CD.B5_38	ERS6577331(SAMEA8894421)	CD.P9s_38	ERS6577591(SAMEA8894682)	D.F2_38	ERS6577851(SAMEA8894942)
S. pimpinellifolium	2018	Peru	Yangas	-11.69946	-76.84604	Site10B	19	healthy	CD.B5_39	ERS6577332(SAMEA8894422)	CD.P9s_39	ERS6577592(SAMEA8894683)	D.F2_39	ERS6577852(SAMEA8894943)
S. habrochaites	2018	Peru	Canta	-11.5130915	-76.678468	Site53	45	infected	CD.B5_40	ERS6577333(SAMEA8894423)	CD.P9s_40	ERS6577593(SAMEA8894684)	D.F2_40	ERS6577853(SAMEA8894944)
S. peruvianum	2018	Peru	Yangas	-11.69946	-76.84604	Site10B	17	healthy	CD.B5_41	ERS6577334(SAMEA8894424)	CD.P9s_41	ERS6577594(SAMEA8894685)	D.F2_41	ERS6577854(SAMEA8894945)
S. habrochaites	2018	Peru	Canta	-11.5130915	-76.678468	Site53	44	infected	CD.B5_42	ERS6577335(SAMEA8894425)	CD.P9s_42	ERS6577595(SAMEA8894686)	D.F2_42	ERS6577855(SAMEA8894946)
S. habrochaites	2018	Peru	Canta	-11.5130915	-76.678468	Site54	52	infected	CD.B5_43	ERS6577336(SAMEA8894426)	CD.P9s_43	ERS6577596(SAMEA8894687)	D.F2_43	ERS6577856(SAMEA8894947)
S. pimpinellifolium	2018	Peru	Yangas	-11.69946	-76.84604	Site10B	19	infected	CD.B5_44	ERS6577337(SAMEA8894427)	CD.P9s_44	ERS6577597(SAMEA8894688)	D.F2_44	ERS6577857(SAMEA8894948)
S. habrochaites	2018	Peru	Canta	-11.5130915	-76.678468	Site53	46	infected	CD.B5_45	ERS6577338(SAMEA8894428)	CD.P9s_45	ERS6577598(SAMEA8894689)	D.F2_45	ERS6577858(SAMEA8894949)
S. habrochaites	2018	Peru	Canta	-11.5130915	-76.678468	Site53	46	infected	CD.B5_46	ERS6577339(SAMEA8894429)	CD.P9s_46	ERS6577599(SAMEA8894690)	D.F2_46	ERS6577859(SAMEA8894950)
S. pimpinellifolium	2018	Peru	Yangas	-11.69946	-76.84604	Site10B	20	healthy	CD.B5_47	ERS6577340(SAMEA8894430)	CD.P9s_47	ERS6577600(SAMEA8894691)	D.F2_47	ERS6577860(SAMEA8894951)
S. habrochaites	2018	Peru	Canta	-11.5130915	-76.678468	Site54	60	healthy	CD.B5_48	ERS6577341(SAMEA8894431)	CD.P9s_48	ERS6577601(SAMEA8894692)	D.F2_48	ERS6577861(SAMEA8894952)
S. peruvianum	2018	Peru	Yangas	-11.69946	-76.84604	Site10B	16	healthy	CD.B5_49	ERS6577342(SAMEA8894432)	CD.P9s_49	ERS6577602(SAMEA8894693)	D.F2_49	ERS6577862(SAMEA8894953)
S. habrochaites	2018	Peru	Canta	-11.4806049	-76.6372699	Site52	39	healthy	CD.B5_50	ERS6577343(SAMEA8894433)	CD.P9s_50	ERS6577603(SAMEA8894694)	D.F2_50	ERS6577863(SAMEA8894954)
S. habrochaites	2018	Peru	Canta	-11.5130915	-76.678468	Site54	51	infected	CD.B5_51	ERS6577344(SAMEA8894434)	CD.P9s_51	ERS6577604(SAMEA8894695)	D.F2_51	ERS6577864(SAMEA8894955)
S. habrochaites	2018	Peru	Canta	-11.5130915	-76.678468	Site54	49	infected	CD.B5_52	ERS6577345(SAMEA8894435)	CD.P9s_52	ERS6577605(SAMEA8894696)	D.F2_52	ERS6577865(SAMEA8894956)
S. habrochaites	2018	Peru	Canta	-11.5130915	-76.678468	Site54	49	healthy	CD.B5_53	ERS6577346(SAMEA8894436)	CD.P9s_53	ERS6577606(SAMEA8894697)	D.F2_53	ERS6577866(SAMEA8894957)
S. habrochaites	2018	Peru	Canta	-11.5130915	-76.678468	Site54	59	healthy	CD.B5_54	ERS6577347(SAMEA8894437)	CD.P9s_54	ERS6577607(SAMEA8894698)	D.F2_54	ERS6577867(SAMEA8894958)
S. pimpinellifolium	2018	Peru	Yangas	-11.69946	-76.84604	Site10B	18	infected	CD.B5_55	ERS6577348(SAMEA8894438)	CD.P9s_55	ERS6577608(SAMEA8894699)	D.F2_55	ERS6577868(SAMEA8894959)
S. habrochaites	2018	Peru	Canta	-11.5130915	-76.678468	Site54	62	healthy	CD.B5_56	ERS6577349(SAMEA8894439)	CD.P9s_56	ERS6577609(SAMEA8894700)	D.F2_56	ERS6577869(SAMEA8894960)
S. habrochaites	2018	Peru	Canta	-11.4806049	-76.6372699	Site52	40	infected	CD.B5_57	ERS6577350(SAMEA8894440)	CD.P9s_57	ERS6577610(SAMEA8894701)	D.F2_57	ERS6577870(SAMEA8894961)
S. habrochaites	2018	Peru	Canta	-11.4806049	-76.6372699	Site52	42	infected	CD.B5_58	ERS6577351(SAMEA8894441)	CD.P9s_58	ERS6577611(SAMEA8894702)	D.F2_58	ERS6577871(SAMEA8894962)
S. habrochaites	2018	Peru	Canta	-11.4806049	-76.6372699	Site52	42	healthy	CD.B5_59	ERS6577352(SAMEA8894442)	CD.P9s_59	ERS6577612(SAMEA8894703)	D.F2_59	ERS6577872(SAMEA8894963)
S. habrochaites	2018	Peru	Canta	-11.5130915	-76.678468	Site53	47	healthy	CD.B5_60	ERS6577353(SAMEA8894443)	CD.P9s_60	ERS6577613(SAMEA8894704)	D.F2_60	ERS6577873(SAMEA8894964)
S. habrochaites	2018	Peru	Canta	-11.4806049	-76.6372699	Site52	37	infected	CD.B5_61	ERS6577354(SAMEA8894444)	CD.P9s_61	ERS6577614(SAMEA8894705)	D.F2_61	ERS6577874(SAMEA8894965)
S. peruvianum	2018	Peru	Yangas	-11.69946	-76.84604	Site10B	16	infected	CD.B5_62	ERS6577355(SAMEA8894445)	CD.P9s_62	ERS6577615(SAMEA8894706)	D.F2_62	ERS6577875(SAMEA8894966)
S. habrochaites	2018	Peru	Canta	-11.4806049	-76.6372699	Site52	37	infected	CD.B5_63	ERS6577356(SAMEA8894446)	CD.P9s_63	ERS6577616(SAMEA8894707)	D.F2_63	ERS6577876(SAMEA8894967)
S. habrochaites	2018	Peru	Canta	-11.4806049	-76.6372699	Site52	36	infected	CD.B5_64	ERS6577357(SAMEA8894447)	CD.P9s_64	ERS6577617(SAMEA8894708)	D.F2_64	ERS6577877(SAMEA8894968)
S. habrochaites	2018	Peru	Canta	-11.4806049	-76.6372699	Site52	38	healthy	CD.B5_65	ERS6577358(SAMEA8894448)	CD.P9s_65	ERS6577618(SAMEA8894709)	D.F2_65	ERS6577878(SAMEA8894969)
S. habrochaites	2018	Peru	Canta	-11.5130915	-76.678468	Site53	44	healthy	CD.B5_66	ERS6577359(SAMEA8894449)	CD.P9s_66	ERS6577619(SAMEA8894710)	D.F2_66	ERS6577879(SAMEA8894970)
S. habrochaites	2018	Peru	Canta	-11.4806049	-76.6372699	Site52	39	infected	CD.B5_67	ERS6577360(SAMEA8894450)	CD.P9s_67	ERS6577620(SAMEA8894711)	D.F2_67	ERS6577880(SAMEA8894971)
S. habrochaites	2018	Peru	Canta	-11.5130915	-76.678468	Site53	47	infected	CD.B5_68	ERS6577361(SAMEA8894451)	CD.P9s_68	ERS6577621(SAMEA8894712)	D.F2_68	ERS6577881(SAMEA8894972)
S. habrochaites	2018	Peru	Canta	-11.4806049	-76.6372699	Site52	40	infected	CD.B5_69	ERS6577362(SAMEA8894452)	CD.P9s_69	ERS6577622(SAMEA8894713)	D.F2_69	ERS6577882(SAMEA8894973)
S. habrochaites	2018	Peru	Canta	-11.5130915	-76.678468	Site54	49	infected	CD.B5_70	ERS6577363(SAMEA8894453)	CD.P9s_70	ERS6577623(SAMEA8894714)	D.F2_70	ERS6577883(SAMEA8894974)
S. habrochaites	2018	Peru	Canta	-11.5130915	-76.678468	Site53	47	infected	CD.B5_71	ERS6577364(SAMEA8894454)	CD.P9s_71	ERS6577624(SAMEA8894715)	D.F2_71	ERS6577884(SAMEA8894975)
S. habrochaites	2018	Peru	Canta	-11.5130915	-76.678468	Site54	49	infected	CD.B5_72	ERS6577365(SAMEA8894455)	CD.P9s_72	ERS6577625(SAMEA8894716)	D.F2_72	ERS6577885(SAMEA8894976)
S. habrochaites	2018	Peru	Canta	-11.5130915	-76.678468	Site53	44	infected	CD.B5_73	ERS6577366(SAMEA8894456)	CD.P9s_73	ERS6577626(SAMEA8894717)	D.F2_73	ERS6577886(SAMEA8894977)
S. habrochaites	2018	Peru	Canta	-11.5130915	-76.678468	Site54	49	healthy	CD.B5_74	ERS6577367(SAMEA8894457)	CD.P9s_74	ERS6577627(SAMEA8894718)	D.F2_74	ERS6577887(SAMEA8894978)
S. habrochaites	2018	Peru	Canta	-11.5130915	-76.678468	Site54	58	healthy	CD.B5_75	ERS6577368(SAMEA8894458)	CD.P9s_75	ERS6577628(SAMEA8894719)	D.F2_75	ERS6577888(SAMEA8894979)
S. habrochaites	2018	Peru	Canta	-11.5130915	-76.678468	Site54	50	infected	CD.B5_76	ERS6577369(SAMEA8894459)	CD.P9s_76	ERS6577629(SAMEA8894720)	D.F2_76	ERS6577889(SAMEA8894980)
S. habrochaites	2018	Peru	Canta	-11.5130915	-76.678468	Site54	53	infected	CD.B5_77	ERS6577370(SAMEA8894460)	CD.P9s_77	ERS6577630(SAMEA8894721)	D.F2_77	ERS6577890(SAMEA8894981)
S. habrochaites	2018	Peru	Canta	-11.5130915	-76.678468	Site54	50	healthy	CD.B5_78	ERS6577371(SAMEA8894461)	CD.P9s_78	ERS6577631(SAMEA8894722)	D.F2_78	ERS6577891(SAMEA8894982)
S. habrochaites	2018	Peru	Canta	-11.5130915	-76.678468	Site54	58	healthy	CD.B5_79	ERS6577372(SAMEA8894462)	CD.P9s_79	ERS6577632(SAMEA8894723)	D.F2_79	ERS6577892(SAMEA8894983)
S. habrochaites	2018	Peru	Canta	-11.5130915	-76.678468	Site54	59	healthy	CD.B5_80	ERS6577373(SAMEA8894463)	CD.P9s_80	ERS6577633(SAMEA8894724)	D.F2_80	ERS6577893(SAMEA8894984)
S. habrochaites	2018	Peru	Canta	-11.5130915	-76.678468	Site54	52	healthy	CD.B5_81	ERS6577374(SAMEA8894464)	CD.P9s_81	ERS6577634(SAMEA8894725)	D.F2_81	ERS6577894(SAMEA8894985)
S. habrochaites	2018	Peru	Canta	-11.5130915	-76.678468	Site54	51	infected	CD.B5_82	ERS6577375(SAMEA8894465)	CD.P9s_82	ERS6577635(SAMEA8894726)	D.F2_82	ERS6577895(SAMEA8894986)
S. habrochaites	2018	Peru	Canta	-11.5130915	-76.678468	Site54	54	infected	CD.B5_83	ERS6577376(SAMEA8894466)	CD.P9s_83	ERS6577636(SAMEA8894727)	D.F2_83	ERS6577896(SAMEA8894987)
S. habrochaites	2018	Peru	Canta	-11.5130915	-76.678468	Site53	43	healthy	CD.B5_84	ERS6577377(SAMEA8894467)	CD.P9s_84	ERS6577637(SAMEA8894728)	D.F2_84	ERS6577897(SAMEA8894988)
S. pimpinellifolium	2018	Peru	Yangas	-11.69946	-76.84604	Site10	1	infected	CD.B5_87	ERS6577378(SAMEA8894468)	CD.P9s_87	ERS6577638(SAMEA8894729)	D.F2_87	ERS6577898(SAMEA8894989)
S. pimpinellifolium	2018	Peru	Yangas	-11.69946	-76.84604	Site10	2	healthy	CD.B5_88	ERS6577379(SAMEA8894469)	CD.P9s_88	ERS6577639(SAMEA8894730)	D.F2_88	ERS6577899(SAMEA8894990)
S. pimpinellifolium	2018	Peru	Yangas	-11.69946	-76.84604	Site10	3	healthy	CD.B5_89	ERS6577380(SAMEA8894470)	CD.P9s_89	ERS6577640(SAMEA8894731)	D.F2_89	ERS6577900(SAMEA8894991)
S. pimpinellifolium	2018	Peru	Yangas	-11.69946	-76.84604	Site10	4	healthy	CD.B5_90	ERS6577381(SAMEA8894471)	CD.P9s_90	ERS6577641(SAMEA8894732)	D.F2_90	ERS6577901(SAMEA8894992)
S. pimpinellifolium	2018	Peru	Yangas	-11.69946	-76.84604	Site10	5	healthy	CD.B5_91	ERS6577382(SAMEA8894472)	CD.P9s_91	ERS6577642(SAMEA8894733)	D.F2_91	ERS6577902(SAMEA8894993)
S. pennellii	2018	Peru	Yangas	-11.69946	-76.84604	Site10	6	infected	CD.B5_92	ERS6577383(SAMEA8894473)	CD.P9s_92	ERS6577643(SAMEA8894734)	D.F2_92	ERS6577903(SAMEA8894994)
S. pennellii	2018	Peru	Yangas	-11.69946	-76.84604	Site10	6	healthy	CD.B5_93	ERS6577384(SAMEA8894474)	CD.P9s_93	ERS6577644(SAMEA8894735)	D.F2_93	ERS6577904(SAMEA8894995)
S. pimpinellifolium	2018	Peru	Yangas	-11.69946	-76.84604	Site10B	7	healthy	CD.B5_94	ERS6577385(SAMEA8894475)	CD.P9s_94	ERS6577645(SAMEA8894736)	D.F2_94	ERS6577905(SAMEA8894996)
S. pimpinellifolium	2018	Peru	Yangas	-11.69946	-76.84604	Site10B	7	infected	CD.B5_95	ERS6577386(SAMEA8894476)	CD.P9s_95	ERS6577646(SAMEA8894737)	D.F2_95	ERS6577906(SAMEA8894997)
S. pimpinellifolium	2018	Peru	Yangas	-11.69946	-76.84604	Site10B	9	infected	CD.B5_97	ERS6577387(SAMEA8894477)	CD.P9s_97	ERS6577647(SAMEA8894738)	D.F2_97	ERS6577907(SAMEA8894998)
S. pimpinellifolium	2018	Peru	Yangas	-11.69946	-76.84604	Site10B	10	healthy	CD.B5_98	ERS6577388(SAMEA8894478)	CD.P9s_98	ERS6577648(SAMEA8894739)	D.F2_98	ERS6577908(SAMEA8894999)
S. pimpinellifolium	2018	Peru	Yangas	-11.69946	-76.84604	Site10B	10	infected	CD.B5_99	ERS6577389(SAMEA8894479)	CD.P9s_99	ERS6577649(SAMEA8894740)	D.F2_99	ERS6577909(SAMEA8895000)
S. pimpinellifolium	2018	Peru	Yangas	-11.69946	-76.84604	Site10B	11	healthy	CD.B5_100	ERS6577390(SAMEA8894480)	CD.P9s_100	ERS6577650(SAMEA8894741)	D.F2_100	ERS6577910(SAMEA8895001)
S. pimpinellifolium	2018	Peru	Yangas	-11.69946	-76.84604	Site10B	11	infected	CD.B5_101	ERS6577391(SAMEA8894481)	CD.P9s_101	ERS6577651(SAMEA8894742)	D.F2_101	ERS6577911(SAMEA8895002)
S. pimpinellifolium	2018	Peru	Yangas	-11.69946	-76.84604	Site10B	12	healthy	CD.B5_102	ERS6577392(SAMEA8894482)	CD.P9s_102	ERS6577652(SAMEA8894743)	D.F2_102	ERS6577912(SAMEA8895003)
S. pimpinellifolium	2018	Peru	Yangas	-11.69946	-76.84604	Site10B	12	infected	CD.B5_103	ERS6577393(SAMEA8894483)	CD.P9s_103	ERS6577653(SAMEA8894744)	D.F2_103	ERS6577913(SAMEA8895004)
S. peruvianum	2018	Peru	Yangas	-11.69946	-76.84604	Site10B	13	healthy	CD.B5_104	ERS6577394(SAMEA8894484)	CD.P9s_104	ERS6577654(SAMEA8894745)	D.F2_104	ERS6577914(SAMEA8895005)
S. peruvianum	2018	Peru	Yangas	-11.69946	-76.84604	Site10B	13	infected	CD.B5_105	ERS6577395(SAMEA8894485)	CD.P9s_105	ERS6577655(SAMEA8894746)	D.F2_105	ERS6577915(SAMEA8895006)
S. peruvianum	2018	Peru	Yangas	-11.69946	-76.84604	Site10B	14	healthy	CD.B5_106	ERS6577396(SAMEA8894486)	CD.P9s_106	ERS6577656(SAMEA8894747)	D.F2_106	ERS6577916(SAMEA8895007)
S. peruvianum	2018	Peru	Yangas	-11.69946	-76.84604	Site10B	14	infected	CD.B5_107	ERS6577397(SAMEA8894487)	CD.P9s_107	ERS6577657(SAMEA8894748)	D.F2_107	ERS6577917(SAMEA8895008)
S. peruvianum	2018	Peru	Yangas	-11.69946	-76.84604	Site10B	15	healthy	CD.B5_108	ERS6577398(SAMEA8894488)	CD.P9s_108	ERS6577658(SAMEA8894749)	D.F2_108	ERS6577918(SAMEA8895009)
S. pimpinellifolium	2018	Peru	Yangas	-11.69946	-76.84604	Site10B	8	healthy	CD.B5_109	ERS6577399(SAMEA8894489)	CD.P9s_109	ERS6577659(SAMEA8894750)	D.F2_109	ERS6577919(SAMEA8895010)
S. pimpinellifolium	2018	Peru	Yangas	-11.69946	-76.84604	Site10B	8	infected	CD.B5_110	ERS6577400(SAMEA8894490)	CD.P9s_110	ERS6577660(SAMEA8894751)	D.F2_110	ERS6577920(SAMEA8895011)
S. pimpinellifolium	2018	Peru	Yangas	-11.69946	-76.84604	Site10B	9	healthy	CD.B5_111	ERS6577401(SAMEA8894491)	CD.P9s_111	ERS6577661(SAMEA8894752)	D.F2_111	ERS6577921(SAMEA8895012)

Host Species	Year	Location	Area	Latitude	Longitude	Site	Plant	State	Bacteria [16S V5]		Eukaryotes [18S V9]		Fungi [ITS2]	
									Library	ENA Accession	Library	ENA Accession	Library	ENA Accession
S. habrochaites	2019	Peru	Canta	-11.457461	-76.626274	3	S3_Plant4	infected	G.B5_1	ERS6577402(SAMEA8894492)	N.P9_1	ERS6577662(SAMEA8894753)	N.F2_1	ERS6577922(SAMEA8895013)
S.corneliomulleri	2019	Peru	Canta	-11.477744	-76.623483	4	S4_Plant3	infected	G.B5_2	ERS6577403(SAMEA8894493)	N.P9_2	ERS6577663(SAMEA8894754)	N.F2_2	ERS6577923(SAMEA8895014)
S.corneliomulleri	2019	Peru	Canta	-11.477744	-76.623483	4	S4_Plant1	healthy	G.B5_3	ERS6577404(SAMEA8894494)	N.P9_3	ERS6577664(SAMEA8894755)	N.F2_3	ERS6577924(SAMEA8895015)
S. habrochaites	2019	Peru	Canta	-11.457461	-76.626274	3	S3_Plant4	infected	G.B5_4	ERS6577405(SAMEA8894495)	N.P9_4	ERS6577665(SAMEA8894756)	N.F2_4	ERS6577925(SAMEA8895016)
S. habrochaites	2019	Peru	Canta	-11.457461	-76.626274	3	S3_Plant1	infected	G.B5_5	ERS6577406(SAMEA8894496)	N.P9_5	ERS6577666(SAMEA8894757)	N.F2_5	ERS6577926(SAMEA8895017)
S.corneliomulleri	2019	Peru	Canta	-11.477744	-76.623483	4	S4_Plant2	healthy	G.B5_6	ERS6577407(SAMEA8894497)	N.P9_6	ERS6577667(SAMEA8894758)	N.F2_6	ERS6577927(SAMEA8895018)
S.corneliomulleri	2019	Peru	Canta	-11.477744	-76.623483	4	S4_Plant5	infected	G.B5_7	ERS6577408(SAMEA8894498)	N.P9_7	ERS6577668(SAMEA8894759)	N.F2_7	ERS6577928(SAMEA8895019)
S.corneliomulleri	2019	Peru	Canta	-11.477744	-76.623483	4	S4_Plant5	infected	G.B5_8	ERS6577409(SAMEA8894499)	N.P9_8	ERS6577669(SAMEA8894760)	N.F2_8	ERS6577929(SAMEA8895020)
S.corneliomulleri	2019	Peru	Canta	-11.477744	-76.623483	4	S4_Plant4	infected	G.B5_9	ERS6577410(SAMEA8894500)	N.P9_9	ERS6577670(SAMEA8894761)	N.F2_9	ERS6577930(SAMEA8895021)
S.corneliomulleri	2019	Peru	Canta	-11.477744	-76.623483	4	S4_Plant4	infected	G.B5_10	ERS6577411(SAMEA8894501)	N.P9_10	ERS6577671(SAMEA8894762)	N.F2_10	ERS6577931(SAMEA8895022)
S. habrochaites	2019	Peru	Canta	-11.457461	-76.626274	3	S3_Plant3	infected	G.B5_11	ERS6577412(SAMEA8894502)	N.P9_11	ERS6577672(SAMEA8894763)	N.F2_11	ERS6577932(SAMEA8895023)
S. habrochaites	2019	Peru	Canta	-11.457461	-76.626274	3	S3_Plant4	infected	G.B5_12	ERS6577413(SAMEA8894503)	N.P9_12	ERS6577673(SAMEA8894764)	N.F2_12	ERS6577933(SAMEA8895024)
S.corneliomulleri	2019	Peru	Canta	-11.477744	-76.623483	4	S4_Plant3	infected	G.B5_13	ERS6577414(SAMEA8894504)	N.P9_13	ERS6577674(SAMEA8894765)	N.F2_13	ERS6577934(SAMEA8895025)
S.corneliomulleri	2019	Peru	Canta	-11.477744	-76.623483	4	S4_Plant2	infected	G.B5_14	ERS6577415(SAMEA8894505)	N.P9_14	ERS6577675(SAMEA8894766)	N.F2_14	ERS6577935(SAMEA8895026)
S. habrochaites	2019	Peru	Canta	-11.457461	-76.626274	3	S3_Plant2	infected	G.B5_15	ERS6577416(SAMEA8894506)	N.P9_15	ERS6577676(SAMEA8894767)	N.F2_15	ERS6577936(SAMEA8895027)
S.corneliomulleri	2019	Peru	Canta	-11.477744	-76.623483	4	S4_Plant1	infected	G.B5_16	ERS6577417(SAMEA8894507)	N.P9_16	ERS6577677(SAMEA8894768)	N.F2_16	ERS6577937(SAMEA8895028)
S.corneliomulleri	2019	Peru	Canta	-11.477744	-76.623483	4	S4_Plant1	healthy	G.B5_17	ERS6577418(SAMEA8894508)	N.P9_17	ERS6577678(SAMEA8894769)	N.F2_17	ERS6577938(SAMEA8895029)
S. habrochaites	2019	Peru	Canta	-11.457461	-76.626274	3	S3_Plant3	infected	G.B5_18	ERS6577419(SAMEA8894509)	N.P9_18	ERS6577679(SAMEA8894770)	N.F2_18	ERS6577939(SAMEA8895030)
S.corneliomulleri	2019	Peru	Canta	-11.477744	-76.623483	4	S4_Plant2	infected	G.B5_19	ERS6577420(SAMEA8894510)	N.P9_19	ERS6577680(SAMEA8894771)	N.F2_19	ERS6577940(SAMEA8895031)
S.corneliomulleri	2019	Peru	Canta	-11.477744	-76.623483	4	S4_Plant4	infected	G.B5_20	ERS6577421(SAMEA8894511)	N.P9_20	ERS6577681(SAMEA8894772)	N.F2_20	ERS6577941(SAMEA8895032)
S.corneliomulleri	2019	Peru	Canta	-11.477744	-76.623483	4	S4_Plant3	healthy	G.B5_21	ERS6577422(SAMEA8894512)	N.P9_21	ERS6577682(SAMEA8894773)	N.F2_21	ERS6577942(SAMEA8895033)
S.corneliomulleri	2019	Peru	Canta	-11.477744	-76.623483	4	S4_Plant2	infected	G.B5_22	ERS6577423(SAMEA8894513)	N.P9_22	ERS6577683(SAMEA8894774)	N.F2_22	ERS6577943(SAMEA8895034)
S. habrochaites	2019	Peru	Canta	-11.457461	-76.626274	3	S3_Plant4	infected	G.B5_23	ERS6577424(SAMEA8894514)	N.P9_23	ERS6577684(SAMEA8894775)	N.F2_23	ERS6577944(SAMEA8895035)
S.corneliomulleri	2019	Peru	Canta	-11.477744	-76.623483	4	S4_Plant1	infected	G.B5_24	ERS6577425(SAMEA8894515)	N.P9_24	ERS6577685(SAMEA8894776)	N.F2_24	ERS6577945(SAMEA8895036)
S. habrochaites	2019	Peru	Canta	-11.457461	-76.626274	3	S3_Plant3	infected	G.B5_25	ERS6577426(SAMEA8894516)	N.P9_25	ERS6577686(SAMEA8894777)	N.F2_25	ERS6577946(SAMEA8895037)
S.corneliomulleri	2019	Peru	Canta	-11.477744	-76.623483	4	S4_Plant3	healthy	G.B5_26	ERS6577427(SAMEA8894517)	N.P9_26	ERS6577687(SAMEA8894778)	N.F2_26	ERS6577947(SAMEA8895038)
S.corneliomulleri	2019	Peru	Canta	-11.477744	-76.623483	4	S4_Plant5	infected	G.B5_27	ERS6577428(SAMEA8894518)	N.P9_27	ERS6577688(SAMEA8894779)	N.F2_27	ERS6577948(SAMEA8895039)
S. habrochaites	2019	Peru	Canta	-11.457461	-76.626274	3	S3_Plant4	infected	G.B5_28	ERS6577429(SAMEA8894519)	N.P9_28	ERS6577689(SAMEA8894780)	N.F2_28	ERS6577949(SAMEA8895040)
S.corneliomulleri	2019	Peru	Canta	-11.477744	-76.623483	4	S4_Plant2	infected	G.B5_29	ERS6577430(SAMEA8894520)	N.P9_29	ERS6577690(SAMEA8894781)	N.F2_29	ERS6577950(SAMEA8895041)
S.corneliomulleri	2019	Peru	Canta	-11.477744	-76.623483	4	S4_Plant2	infected	G.B5_30	ERS6577431(SAMEA8894521)	N.P9_30	ERS6577691(SAMEA8894782)	N.F2_30	ERS6577951(SAMEA8895042)
S. habrochaites	2019	Peru	Canta	-11.457461	-76.626274	3	S3_Plant1	healthy	G.B5_31	ERS6577432(SAMEA8894522)	N.P9_31	ERS6577692(SAMEA8894783)	N.F2_31	ERS6577952(SAMEA8895043)
S.corneliomulleri	2019	Peru	Canta	-11.448796	-76.621971	2	S2_Plant2	infected	G.B5_32	ERS6577433(SAMEA8894523)	N.P9_32	ERS6577693(SAMEA8894784)	N.F2_32	ERS6577953(SAMEA8895044)
S.corneliomulleri	2019	Peru	Canta	-11.477744	-76.623483	4	S4_Plant2	infected	G.B5_33	ERS6577434(SAMEA8894524)	N.P9_33	ERS6577694(SAMEA8894785)	N.F2_33	ERS6577954(SAMEA8895045)
S.corneliomulleri	2019	Peru	Canta	-11.477744	-76.623483	4	S4_Plant4	infected	G.B5_34	ERS6577435(SAMEA8894525)	N.P9_34	ERS6577695(SAMEA8894786)	N.F2_34	ERS6577955(SAMEA8895046)
S.corneliomulleri	2019	Peru	Canta	-11.448796	-76.621971	2	S2_Plant2	healthy	G.B5_35	ERS6577436(SAMEA8894526)	N.P9_35	ERS6577696(SAMEA8894787)	N.F2_35	ERS6577956(SAMEA8895047)
S. habrochaites	2019	Peru	Canta	-11.457461	-76.626274	3	S3_Plant3	infected	G.B5_36	ERS6577437(SAMEA8894527)	N.P9_36	ERS6577697(SAMEA8894788)	N.F2_36	ERS6577957(SAMEA8895048)
S.corneliomulleri	2019	Peru	Canta	-11.448796	-76.621971	2	S2_Plant3	healthy	G.B5_37	ERS6577438(SAMEA8894528)	N.P9_37	ERS6577698(SAMEA8894789)	N.F2_37	ERS6577958(SAMEA8895049)
S.corneliomulleri	2019	Peru	Canta	-11.477744	-76.623483	4	S4_Plant3	healthy	G.B5_38	ERS6577439(SAMEA8894529)	N.P9_38	ERS6577699(SAMEA8894790)	N.F2_38	ERS6577959(SAMEA8895050)
S.corneliomulleri	2019	Peru	Canta	-11.453299	-76.623854	1	S1_Plant4	healthy	G.B5_39	ERS6577440(SAMEA8894530)	N.P9_39	ERS6577700(SAMEA8894791)	N.F2_39	ERS6577960(SAMEA8895051)
S.corneliomulleri	2019	Peru	Canta	-11.448796	-76.621971	2	S2_Plant3	infected	G.B5_40	ERS6577441(SAMEA8894531)	N.P9_40	ERS6577701(SAMEA8894792)	N.F2_40	ERS6577961(SAMEA8895052)
S.corneliomulleri	2019	Peru	Canta	-11.477744	-76.623483	4	S4_Plant4	healthy	G.B5_41	ERS6577442(SAMEA8894532)	N.P9_41	ERS6577702(SAMEA8894793)	N.F2_41	ERS6577962(SAMEA8895053)
S. habrochaites	2019	Peru	Canta	-11.457461	-76.626274	3	S3_Plant4	infected	G.B5_42	ERS6577443(SAMEA8894533)	N.P9_42	ERS6577703(SAMEA8894794)	N.F2_42	ERS6577963(SAMEA8895054)
S.corneliomulleri	2019	Peru	Canta	-11.477744	-76.623483	4	S4_Plant5	infected	G.B5_43	ERS6577444(SAMEA8894534)	N.P9_43	ERS6577704(SAMEA8894795)	N.F2_43	ERS6577964(SAMEA8895055)
S. habrochaites	2019	Peru	Canta	-11.457461	-76.626274	3	S3_Plant4	healthy	G.B5_44	ERS6577445(SAMEA8894535)	N.P9_44	ERS6577705(SAMEA8894796)	N.F2_44	ERS6577965(SAMEA8895056)
S.corneliomulleri	2019	Peru	Canta	-11.453299	-76.623854	1	S1_Plant5	infected	G.B5_45	ERS6577446(SAMEA8894536)	N.P9_45	ERS6577706(SAMEA8894797)	N.F2_45	ERS6577966(SAMEA8895057)
S.corneliomulleri	2019	Peru	Canta	-11.448796	-76.621971	2	S2_Plant3	infected	G.B5_46	ERS6577447(SAMEA8894537)	N.P9_46	ERS6577707(SAMEA8894798)	N.F2_46	ERS6577967(SAMEA8895058)
S. habrochaites	2019	Peru	Canta	-11.457461	-76.626274	3	S3_Plant5	infected	G.B5_47	ERS6577448(SAMEA8894538)	N.P9_47	ERS6577708(SAMEA8894799)	N.F2_47	ERS6577968(SAMEA8895059)
S.corneliomulleri	2019	Peru	Canta	-11.477744	-76.623483	4	S4_Plant4	healthy	G.B5_48	ERS6577449(SAMEA8894539)	N.P9_48	ERS6577709(SAMEA8894800)	N.F2_48	ERS6577969(SAMEA8895060)
S. habrochaites	2019	Peru	Canta	-11.457461	-76.626274	3	S3_Plant3	infected	G.B5_49	ERS6577450(SAMEA8894540)	N.P9_49	ERS6577710(SAMEA8894801)	N.F2_49	ERS6577970(SAMEA8895061)
S.corneliomulleri	2019	Peru	Canta	-11.448796	-76.621971	2	S2_Plant1	infected	G.B5_50	ERS6577451(SAMEA8894541)	N.P9_50	ERS6577711(SAMEA8894802)	N.F2_50	ERS6577971(SAMEA8895062)
S. habrochaites	2019	Peru	Canta	-11.457461	-76.626274	3	S3_Plant2	infected	G.B5_51	ERS6577452(SAMEA8894542)	N.P9_51	ERS6577712(SAMEA8894803)	N.F2_51	ERS6577972(SAMEA8895063)
S.corneliomulleri	2019	Peru	Canta	-11.477744	-76.623483	4	S4_Plant1	infected	G.B5_52	ERS6577453(SAMEA8894543)	N.P9_52	ERS6577713(SAMEA8894804)	N.F2_52	ERS6577973(SAMEA8895064)
S.corneliomulleri	2019	Peru	Canta	-11.453299	-76.623854	1	S1_Plant5	healthy	G.B5_53	ERS6577454(SAMEA8894544)	N.P9_53	ERS6577714(SAMEA8894805)	N.F2_53	ERS6577974(SAMEA8895065)
S.corneliomulleri	2019	Peru	Canta	-11.453299	-76.623854	1	S1_Plant5	healthy	G.B5_54	ERS6577455(SAMEA8894545)	N.P9_54	ERS6577715(SAMEA8894806)	N.F2_54	ERS6577975(SAMEA8895066)
S.corneliomulleri	2019	Peru	Canta	-11.453299	-76.623854	1	S1_Plant4	infected	G.B5_55	ERS6577456(SAMEA8894546)	N.P9_55	ERS6577716(SAMEA8894807)	N.F2_55	ERS6577976(SAMEA8895067)
S. habrochaites	2019	Peru	Canta	-11.457461	-76.626274	3	S3_Plant5	infected	G.B5_56	ERS6577457(SAMEA8894547)	N.P9_56	ERS6577717(SAMEA8894808)	N.F2_56	ERS6577977(SAMEA8895068)
S.corneliomulleri	2019	Peru	Canta	-11.453299	-76.623854	1	S1_Plant2	infected	G.B5_57	ERS6577458(SAMEA8894548)	N.P9_57	ERS6577718(SAMEA8894809)	N.F2_57	ERS6577978(SAMEA8895069)
S.corneliomulleri	2019	Peru	Canta	-11.477744	-76.623483	4	S4_Plant3	infected	G.B5_58	ERS6577459(SAMEA8894549)	N.P9_58	ERS6577719(SAMEA8894810)	N.F2_58	ERS6577979(SAMEA8895070)
S.corneliomulleri	2019	Peru	Canta	-11.453299	-76.623854	1	S1_Plant4	infected	G.B5_59	ERS6577460(SAMEA8894550)	N.P9_59	ERS6577720(SAMEA8894811)	N.F2_59	ERS6577980(SAMEA8895071)
S.corneliomulleri	2019	Peru	Canta	-11.448796	-76.621971	2	S2_Plant1	healthy	G.B5_60	ERS6577461(SAMEA8894551)	N.P9_60	ERS6577721(SAMEA8894812)	N.F2_60	ERS6577981(SAMEA8895072)
S.corneliomulleri	2019	Peru	Canta	-11.448796	-76.621971	2	S2_Plant2	infected	G.B5_61	ERS6577462(SAMEA8894552)	N.P9_61	ERS6577722(SAMEA8894813)	N.F2_61	ERS6577982(SAMEA8895073)
S.corneliomulleri	2019	Peru	Canta	-11.453299	-76.623854	1	S1_Plant1	healthy	G.B5_62	ERS6577463(SAMEA8894553)	N.P9_62	ERS6577723(SAMEA8894814)	N.F2_62	ERS6577983(SAMEA8895074)
S.corneliomulleri	2019	Peru	Canta	-11.477744	-76.623483	4	S4_Plant3	infected	G.B5_63	ERS6577464(SAMEA8894554)	N.P9_63	ERS6577724(SAMEA8894815)	N.F2_63	ERS6577984(SAMEA8895075)
S.corneliomulleri	2019	Peru	Canta	-11.453299	-76.623854	1	S1_Plant3	infected	G.B5_64	ERS6577465(SAMEA8894555)	N.P9_64	ERS6577725(SAMEA8894816)	N.F2_64	ERS6577985(SAMEA8895076)
S.corneliomulleri	2019	Peru	Canta	-11.477744	-76.623483	4	S4_Plant3	infected	G.B5_65	ERS6577466(SAMEA8894556)	N.P9_65	ERS6577726(SAMEA8894817)	N.F2_65	ERS6577986(SAMEA8895077)
S.corneliomulleri	2019	Peru	Canta	-11.448796	-76.621971	2	S2_Plant4	infected	G.B5_66	ERS6577467(SAMEA8894557)	N.P9_66	ERS6577727(SAMEA8894818)	N.F2_66	ERS6577987(SAMEA8895078)
S.corneliomulleri	2019	Peru	Canta	-11.477744	-76.623483	4	S4_Plant5	infected	G.B5_67	ERS6577468(SAMEA8894558)	N.P9_67	ERS6577728(SAMEA8894819)	N.F2_67	ERS6577988(SAMEA8895079)
S.corneliomulleri	2019	Peru	Canta	-11.477744	-76.623483	4	S4_Plant5	healthy	G.B5_68	ERS6577469(SAMEA8894559)	N.P9_68	ERS6577729(SAMEA8894820)	N.F2_68	ERS6577989(SAMEA8895080)
S.corneliomulleri	2019	Peru	Canta	-11.453299	-76.623854	1	S1_Plant3	healthy	G.B5_69	ERS6577470(SAMEA8894560)	N.P9_69	ERS6577730(SAMEA8894821)	N.F2_69	ERS6577990(SAMEA8895081)
S.corneliomulleri	2019	Peru	Canta	-11.453299	-76.623854	1	S1_Plant5	healthy	G.B5_70	ERS6577471(SAMEA8894561)	N.P9_70	ERS6577731(SAMEA8894822)	N.F2_70	ERS6577991(SAMEA8895082)
S.corneliomulleri	2019	Peru	Canta	-11.477744	-76.623483	4	S4_Plant1	healthy	G.B5_71	ERS6577472(SAMEA8894562)	N.P9_71	ERS6577732(SAMEA8894823)	N.F2_71	ERS6577992(SAMEA8895083)
S.corneliomulleri	2019	Peru	Canta	-11.448796	-76.621971	2	S2_Plant3	infected	G.B5_72	ERS6577473(SAMEA8894563)	N.P9_72	ERS6577733(SAMEA8894824)	N.F2_72	ERS6577993(SAMEA8895084)
S.corneliomulleri	2019	Peru	Canta	-11.477744	-76.623483	4	S4_Plant2	healthy	G.B5_73	ERS6577474(SAMEA8894564)	N.P9_73	ERS6577734(SAMEA8894825)	N.F2_73	ERS6577994(SAMEA8895085)
S. habrochaites	2019	Peru	Canta	-11.457461	-76.626274	3	S3_Plant5	infected	G.B5_74	ERS6577475(SAMEA8894565)	N.P9_74	ERS6577735(SAMEA8894826)	N.F2_74	ERS6577995(SAMEA8895086)
S.corneliomulleri	2019	Peru	Canta	-11.477744	-76.623483	4	S4_Plant4	infected	G.B5_75	ERS6577476(SAMEA8894566)	N.P9_75	ERS6577736(SAMEA8894827)	N.F2_75	ERS6577996(SAMEA8895087)
S.corneliomulleri	2019	Peru	Canta	-11.477744	-76.623483	4	S4_Plant4	infected	G.B5_76	ERS6577477(SAMEA8894567)	N.P9_76	ERS6577737(SAMEA8894828)	N.F2_76	ERS6577997(SAMEA8895088)
S.corneliomulleri	2019	Peru	Canta	-11.477744	-76.623483	4	S4_Plant1	infected	G.B5_77	ERS6577478(SAMEA8894568)	N.P9_77	ERS6577738(SAMEA8894829)	N.F2_77	ERS6577998(SAMEA8895089)
S. habrochaites	2019	Peru	Canta	-11.457461	-76.626274	3	S3_Plant1	infected	G.B5_78	ERS6577479(SAMEA8894569)	N.P9_78	ERS6577739(SAMEA8894830)	N.F2_78	ERS6577999(SAMEA8895090)
S. habrochaites	2019	Peru	Canta	-11.457461	-76.626274	3	S3_Plant5	infected	G.B5_79	ERS6577480(SAMEA8894570)	N.P9_79	ERS6577740(SAMEA8894831)	N.F2_79	ERS6578000(SAMEA8895091)
S.corneliomulleri	2019	Peru	Canta	-11.477744	-76.623483	4	S4_Plant5	healthy	G.B5_80	ERS6577481(SAMEA8894571)	N.P9_80	ERS6577741(SAMEA8894832)	N.F2_80	ERS6578001(SAMEA8895092)
S. habrochaites	2019	Peru	Canta	-11.457461	-76.626274	3	S3_Plant1	infected	G.B5_81	ERS6577482(SAMEA8894572)	N.P9_81	ERS6577742(SAMEA8894833)	N.F2_81	ERS6578002(SAMEA8895093)
S.corneliomulleri	2019	Peru	Canta	-11.477744	-76.623483	4	S4_Plant3	infected	G.B5_82	ERS6577483(SAMEA8894573)	N.P9_82	ERS6577743(SAMEA8894834)	N.F2_82	ERS6578003(SAMEA8895094)
S. habrochaites	2019	Peru	Canta	-11.457461	-76.626274	3	S3_Plant2	infected	G.B5_83	ERS6577484(SAMEA8894574)	N.P9_83	ERS6577744(SAMEA8894835)	N.F2_83	ERS6578004(SAMEA8895095)
S.corneliomulleri	2019	Peru	Canta	-11.477744	-76.623483	4	S4_Plant5	infected	G.B5_84	ERS6577485(SAMEA8894575)	N.P9_84	ERS6577745(SAMEA8894836)	N.F2_84	ERS6578005(SAMEA8895096)
S.corneliomulleri	2019	Peru	Canta	-11.453299	-76.623854	1	S1_Plant4	healthy	G.B5_85	ERS6577486(SAMEA8894576)	N.P9_85	ERS6577746(SAMEA8894837)	N.F2_85	ERS6578006(SAMEA8895097)
S. habrochaites	2019	Peru	Canta	-11.457461	-76.626274	3	S3_Plant2	infected	G.B5_86	ERS6577487(SAMEA8894577)	N.P9_86	ERS6577747(SAMEA8894838)	N.F2_86	ERS6578007(SAMEA8895098)
S. habrochaites	2019	Peru	Canta	-11.457461	-76.626274	3	S3_Plant5	infected	G.B5_87	ERS6577488(SAMEA8894578)	N.P9_87	ERS6577748(SAMEA8894839)	N.F2_87	ERS6578008(SAMEA8895099)
S.corneliomulleri	2019	Peru	Canta	-11.453299	-76.623854	1	S1_Plant1	healthy	G.B5_88	ERS6577489(SAMEA8894579)	N.P9_88	ERS6577749(SAMEA8894840)	N.F2_88	ERS6578009(SAMEA8895100)
S.corneliomulleri	2019	Peru	Canta	-11.477744	-76.623483	4	S4_Plant5	healthy	G.B5_89	ERS6577490(SAMEA8894580)	N.P9_89	ERS6577750(SAMEA8894841)	N.F2_89	ERS6578010(SAMEA8895101)
S.corneliomulleri	2019	Peru	Canta	-11.477744	-76.623483	4	S4_Plant1	infected	G.B5_90	ERS6577491(SAMEA8894581)	N.P9_90	ERS6577751(SAMEA8894842)	N.F2_90	ERS6578011(SAMEA8895102)
S.corneliomulleri	2019	Peru	Canta	-11.448796	-76.621971	2	S2_Plant4	healthy	G.B5_91	ERS6577492(SAMEA8894582)	N.P9_91	ERS6577752(SAMEA8894843)	N.F2_91	ERS6578012(SAMEA8895103)
S.corneliomulleri	2019	Peru	Canta	-11.448796	-76.621971	2	S2_Plant1	healthy	G.B5_92	ERS6577493(SAMEA8894583)	N.P9_92	ERS6577753(SAMEA8894844)	N.F2_92	ERS6578013(SAMEA8895104)
S.corneliomulleri	2019	Peru	Canta	-11.448796	-76.621971	2	S2_Plant4	healthy	G.B5_93	ERS6577494(SAMEA8894584)	N.P9_93	ERS6577754(SAMEA8894845)	N.F2_93	ERS6578014(SAMEA8895105)
S.corneliomulleri	2019	Peru	Canta	-11.448796	-76.621971	2	S2_Plant2	healthy	G.B5_94	ERS6577495(SAMEA8894585)	N.P9_94	ERS6577755(SAMEA8894846)	N.F2_94	ERS6578015(SAMEA8895106)
S. habrochaites	2019	Peru	Canta	-11.457461	-76.626274	3	S3_Plant4	healthy	G.B5_95	ERS6577496(SAMEA8894586)	N.P9_95	ERS6577756(SAMEA8894847)	N.F2_95	ERS6578016(SAMEA8895107)
S. habrochaites	2019	Peru	Canta	-11.457461	-76.626274	3	S3_Plant2	healthy	G.B5_97	ERS6577497(SAMEA8894587)	N.P9_97	ERS6577757(SAMEA8894848)	N.F2_97	ERS6578017(SAMEA8895108)
S.corneliomulleri	2019	Peru	Canta	-11.453299	-76.623854	1	S1_Plant3	infected	G.B5_98	ERS6577498(SAMEA8894588)	N.P9_98	ERS6577758(SAMEA8894849)	N.F2_98	ERS6578018(SAMEA8895109)
S.corneliomulleri	2019	Peru	Canta	-11.453299	-76.623854	1	S1_Plant2	infected	G.B5_99	ERS6577499(SAMEA8894589)	N.P9_99	ERS6577759(SAMEA8894850)	N.F2_99	ERS6578019(SAMEA8895110)
S.corneliomulleri	2019	Peru	Canta	-11.448796	-76.621971	2	S2_Plant2	healthy	G.B5_100	ERS6577500(SAMEA8894590)	N.P9_100	ERS6577760(SAMEA8894851)	N.F2_100	ERS6578020(SAMEA8895111)
S. habrochaites	2019	Peru	Canta	-11.457461	-76.626274	3	S3_Plant1	infected	G.B5_101	ERS6577501(SAMEA8894591)	N.P9_101	ERS6577761(SAMEA8894852)	N.F2_101	ERS6578021(SAMEA8895112)
S.corneliomulleri	2019	Peru	Canta	-11.453299	-76.623854	1	S1_Plant5	infected	G.B5_102	ERS6577502(SAMEA8894592)	N.P9_102	ERS6577762(SAMEA8894853)	N.F2_102	ERS6578022(SAMEA8895113)
S.corneliomulleri	2019	Peru	Canta	-11.448796	-76.621971	2	S2_Plant3	healthy	G.B5_103	ERS6577503(SAMEA8894593)	N.P9_103	ERS6577763(SAMEA8894854)	N.F2_103	ERS6578023(SAMEA8895114)
S.corneliomulleri	2019	Peru	Canta	-11.453299	-76.623854	1	S1_Plant4	healthy	G.B5_104	ERS6577504(SAMEA8894594)	N.P9_104	ERS6577764(SAMEA8894855)	N.F2_104	ERS6578024(SAMEA8895115)
S.corneliomulleri	2019	Peru	Canta	-11.453299	-76.623854	1	S1_Plant3	infected	G.B5_105	ERS6577505(SAMEA8894595)	N.P9_105	ERS6577765(SAMEA8894856)	N.F2_105	ERS6578025(SAMEA8895116)
S.corneliomulleri	2019	Peru	Canta	-11.453299	-76.623854	1	S1_Plant3	healthy	G.B5_106	ERS6577506(SAMEA8894596)	N.P9_106	ERS6577766(SAMEA8894857)	N.F2_106	ERS6578026(SAMEA8895117)
S.corneliomulleri	2019	Peru	Canta	-11.448796	-76.621971	2	S2_Plant5	infected	G.B5_107	ERS6577507(SAMEA8894597)	N.P9_107	ERS6577767(SAMEA8894858)	N.F2_107	ERS6578027(SAMEA8895118)
S. habrochaites	2019	Peru	Canta	-11.457461	-76.626274	3	S3_Plant5	healthy	G.B5_108	ERS6577508(SAMEA8894598)	N.P9_108	ERS6577768(SAMEA8894859)	N.F2_108	ERS6578028(SAMEA8895119)
S.corneliomulleri	2019	Peru	Canta	-11.448796	-76.621971	2	S2_Plant4	infected	G.B5_109	ERS6577509(SAMEA8894599)	N.P9_109	ERS6577769(SAMEA8894860)	N.F2_109	ERS6578029(SAMEA8895120)
S. habrochaites	2019	Peru	Canta	-11.457461	-76.626274	3	S3_Plant3	infected	G.B5_110	ERS6577510(SAMEA8894600)	N.P9_110	ERS6577770(SAMEA8894861)	N.F2_110	ERS6578030(SAMEA8895121)
S.corneliomulleri	2019	Peru	Canta	-11.453299	-76.623854	1	S1_Plant5	infected	G.B5_111	ERS6577511(SAMEA8894601)	N.P9_111	ERS6577771(SAMEA8894862)	N.F2_111	ERS6578031(SAMEA8895122)

Host Species	Year	Location	Area	Latitude	Longitude	Site	Plant	State	Bacteria [16S V5]		Eukaryotes [18S V9]		Fungi [ITS2]	
									Library	ENA Accession	Library	ENA Accession	Library	ENA Accession
S. corneliomulleri	2019	Peru	Canta	-11.453299	-76.623854	1	S1_Plant2	healthy	G.B5_112	ERS6577512(SAMEA8894602)	N.P9_112	ERS6577772(SAMEA8894863)	N.F2_112	ERS6578032(SAMEA8895123)
S. corneliomulleri	2019	Peru	Canta	-11.448796	-76.621971	2	S2_Plant5	healthy	G.B5_113	ERS6577513(SAMEA8894603)	N.P9_113	ERS6577773(SAMEA8894864)	N.F2_113	ERS6578033(SAMEA8895124)
S. habrochaites	2019	Peru	Canta	-11.457461	-76.626274	3	S3_Plant1	infected	G.B5_114	ERS6577514(SAMEA8894604)	N.P9_114	ERS6577774(SAMEA8894865)	N.F2_114	ERS6578034(SAMEA8895125)
S. corneliomulleri	2019	Peru	Canta	-11.448796	-76.621971	2	S2_Plant1	healthy	G.B5_115	ERS6577515(SAMEA8894605)	N.P9_115	ERS6577775(SAMEA8894866)	N.F2_115	ERS6578035(SAMEA8895126)
S. corneliomulleri	2019	Peru	Canta	-11.448796	-76.621971	2	S2_Plant4	infected	G.B5_116	ERS6577516(SAMEA8894606)	N.P9_116	ERS6577776(SAMEA8894867)	N.F2_116	ERS6578036(SAMEA8895127)
S. corneliomulleri	2019	Peru	Canta	-11.448796	-76.621971	2	S2_Plant5	infected	G.B5_117	ERS6577517(SAMEA8894607)	N.P9_117	ERS6577777(SAMEA8894868)	N.F2_117	ERS6578037(SAMEA8895128)
S. corneliomulleri	2019	Peru	Canta	-11.453299	-76.623854	1	S1_Plant1	infected	G.B5_118	ERS6577518(SAMEA8894608)	N.P9_118	ERS6577778(SAMEA8894869)	N.F2_118	ERS6578038(SAMEA8895129)
S. corneliomulleri	2019	Peru	Canta	-11.453299	-76.623854	1	S1_Plant2	healthy	G.B5_119	ERS6577519(SAMEA8894609)	N.P9_119	ERS6577779(SAMEA8894870)	N.F2_119	ERS6578039(SAMEA8895130)
S. corneliomulleri	2019	Peru	Canta	-11.448796	-76.621971	2	S2_Plant5	infected	G.B5_120	ERS6577520(SAMEA8894610)	N.P9_120	ERS6577780(SAMEA8894871)	N.F2_120	ERS6578040(SAMEA8895131)
S. habrochaites	2019	Peru	Canta	-11.457461	-76.626274	3	S3_Plant3	healthy	G.B5_121	ERS6577521(SAMEA8894611)	N.P9_121	ERS6577781(SAMEA8894872)	N.F2_121	ERS6578041(SAMEA8895132)
S. corneliomulleri	2019	Peru	Canta	-11.453299	-76.623854	1	S1_Plant3	healthy	G.B5_122	ERS6577522(SAMEA8894612)	N.P9_122	ERS6577782(SAMEA8894873)	N.F2_122	ERS6578042(SAMEA8895133)
S. habrochaites	2019	Peru	Canta	-11.457461	-76.626274	3	S3_Plant2	infected	G.B5_123	ERS6577523(SAMEA8894613)	N.P9_123	ERS6577783(SAMEA8894874)	N.F2_123	ERS6578043(SAMEA8895134)
S. corneliomulleri	2019	Peru	Canta	-11.448796	-76.621971	2	S2_Plant3	healthy	G.B5_124	ERS6577524(SAMEA8894614)	N.P9_124	ERS6577784(SAMEA8894875)	N.F2_124	ERS6578044(SAMEA8895135)
S. corneliomulleri	2019	Peru	Canta	-11.453299	-76.623854	1	S1_Plant2	healthy	G.B5_125	ERS6577525(SAMEA8894615)	N.P9_125	ERS6577785(SAMEA8894876)	N.F2_125	ERS6578045(SAMEA8895136)
S. habrochaites	2019	Peru	Canta	-11.457461	-76.626274	3	S3_Plant3	healthy	G.B5_126	ERS6577526(SAMEA8894616)	N.P9_126	ERS6577786(SAMEA8894877)	N.F2_126	ERS6578046(SAMEA8895137)
S. habrochaites	2019	Peru	Canta	-11.457461	-76.626274	3	S3_Plant2	infected	G.B5_127	ERS6577527(SAMEA8894617)	N.P9_127	ERS6577787(SAMEA8894878)	N.F2_127	ERS6578047(SAMEA8895138)
S. habrochaites	2019	Peru	Canta	-11.457461	-76.626274	3	S3_Plant1	healthy	G.B5_128	ERS6577528(SAMEA8894618)	N.P9_128	ERS6577788(SAMEA8894879)	N.F2_128	ERS6578048(SAMEA8895139)
S. corneliomulleri	2019	Peru	Canta	-11.477744	-76.623483	4	S4_Plant1	infected	G.B5_129	ERS6577529(SAMEA8894619)	N.P9_129	ERS6577789(SAMEA8894880)	N.F2_129	ERS6578049(SAMEA8895140)
S. corneliomulleri	2019	Peru	Canta	-11.453299	-76.623854	1	S1_Plant4	infected	G.B5_130	ERS6577530(SAMEA8894620)	N.P9_130	ERS6577790(SAMEA8894881)	N.F2_130	ERS6578050(SAMEA8895141)
S. corneliomulleri	2019	Peru	Canta	-11.448796	-76.621971	2	S2_Plant1	infected	G.B5_131	ERS6577531(SAMEA8894621)	N.P9_131	ERS6577791(SAMEA8894882)	N.F2_131	ERS6578051(SAMEA8895142)
S. corneliomulleri	2019	Peru	Canta	-11.448796	-76.621971	2	S2_Plant4	healthy	G.B5_132	ERS6577532(SAMEA8894622)	N.P9_132	ERS6577792(SAMEA8894883)	N.F2_132	ERS6578052(SAMEA8895143)
S. corneliomulleri	2019	Peru	Canta	-11.453299	-76.623854	1	S1_Plant1	infected	G.B5_133	ERS6577533(SAMEA8894623)	N.P9_133	ERS6577793(SAMEA8894884)	N.F2_133	ERS6578053(SAMEA8895144)
S. corneliomulleri	2019	Peru	Canta	-11.453299	-76.623854	1	S1_Plant1	infected	G.B5_134	ERS6577534(SAMEA8894624)	N.P9_134	ERS6577794(SAMEA8894885)	N.F2_134	ERS6578054(SAMEA8895145)
S. habrochaites	2019	Peru	Canta	-11.457461	-76.626274	3	S3_Plant2	healthy	G.B5_135	ERS6577535(SAMEA8894625)	N.P9_135	ERS6577795(SAMEA8894886)	N.F2_135	ERS6578055(SAMEA8895146)
S. habrochaites	2019	Peru	Canta	-11.457461	-76.626274	3	S3_Plant5	healthy	G.B5_136	ERS6577536(SAMEA8894626)	N.P9_136	ERS6577796(SAMEA8894887)	N.F2_136	ERS6578056(SAMEA8895147)
S. habrochaites	2019	Peru	Canta	-11.457461	-76.626274	3	S3_Plant3	healthy	G.B5_137	ERS6577537(SAMEA8894627)	N.P9_137	ERS6577797(SAMEA8894888)	N.F2_137	ERS6578057(SAMEA8895148)
S. habrochaites	2019	Peru	Canta	-11.457461	-76.626274	3	S3_Plant2	healthy	G.B5_138	ERS6577538(SAMEA8894628)	N.P9_138	ERS6577798(SAMEA8894889)	N.F2_138	ERS6578058(SAMEA8895149)
S. corneliomulleri	2019	Peru	Canta	-11.477744	-76.623483	4	S4_Plant4	healthy	G.B5_139	ERS6577539(SAMEA8894629)	N.P9_139	ERS6577799(SAMEA8894890)	N.F2_139	ERS6578059(SAMEA8895150)
S. habrochaites	2019	Peru	Canta	-11.457461	-76.626274	3	S3_Plant5	infected	G.B5_140	ERS6577540(SAMEA8894630)	N.P9_140	ERS6577800(SAMEA8894891)	N.F2_140	ERS6578060(SAMEA8895151)
S. corneliomulleri	2019	Peru	Canta	-11.448796	-76.621971	2	S2_Plant5	healthy	G.B5_141	ERS6577541(SAMEA8894631)	N.P9_141	ERS6577801(SAMEA8894892)	N.F2_141	ERS6578061(SAMEA8895152)
S. habrochaites	2019	Peru	Canta	-11.457461	-76.626274	3	S3_Plant1	healthy	G.B5_142	ERS6577542(SAMEA8894632)	N.P9_142	ERS6577802(SAMEA8894893)	N.F2_142	ERS6578062(SAMEA8895153)
S. corneliomulleri	2019	Peru	Canta	-11.453299	-76.623854	1	S1_Plant2	infected	G.B5_143	ERS6577543(SAMEA8894633)	N.P9_143	ERS6577803(SAMEA8894894)	N.F2_143	ERS6578063(SAMEA8895154)
S. corneliomulleri	2019	Peru	Canta	-11.448796	-76.621971	2	S2_Plant2	infected	G.B5_144	ERS6577544(SAMEA8894634)	N.P9_144	ERS6577804(SAMEA8894895)	N.F2_144	ERS6578064(SAMEA8895155)
S. corneliomulleri	2019	Peru	Canta	-11.477744	-76.623483	4	S4_Plant2	healthy	G.B5_145	ERS6577545(SAMEA8894635)	N.P9_145	ERS6577805(SAMEA8894896)	N.F2_145	ERS6578065(SAMEA8895156)
S. habrochaites	2019	Peru	Canta	-11.457461	-76.626274	3	S3_Plant5	healthy	G.B5_146	ERS6577546(SAMEA8894636)	N.P9_146	ERS6577806(SAMEA8894897)	N.F2_146	ERS6578066(SAMEA8895157)
S. habrochaites	2019	Peru	Canta	-11.457461	-76.626274	3	S3_Plant1	infected	G.B5_147	ERS6577547(SAMEA8894637)	N.P9_147	ERS6577807(SAMEA8894898)	N.F2_147	ERS6578067(SAMEA8895158)
S. habrochaites	2019	Peru	Canta	-11.457461	-76.626274	3	S3_Plant4	healthy	G.B5_148	ERS6577548(SAMEA8894638)	N.P9_148	ERS6577808(SAMEA8894899)	N.F2_148	ERS6578068(SAMEA8895159)
S. corneliomulleri	2019	Peru	Canta	-11.448796	-76.621971	2	S2_Plant1	infected	G.B5_149	ERS6577549(SAMEA8894639)	N.P9_149	ERS6577809(SAMEA8894900)	N.F2_149	ERS6578069(SAMEA8895160)
S. corneliomulleri	2019	Peru	Canta	-11.448796	-76.621971	2	S2_Plant5	healthy	G.B5_150	ERS6577550(SAMEA8894640)	N.P9_150	ERS6577810(SAMEA8894901)	N.F2_150	ERS6578070(SAMEA8895161)
S. corneliomulleri	2019	Peru	Canta	-11.453299	-76.623854	1	S1_Plant1	healthy	G.B5_151	ERS6577551(SAMEA8894641)	N.P9_151	ERS6577811(SAMEA8894902)	N.F2_151	ERS6578071(SAMEA8895162)
S. habrochaites	2019	Peru	Canta	-11.457461	-76.626274	3	S3_Plant4	infected	G.B5_152	ERS6577552(SAMEA8894642)	N.P9_152	ERS6577812(SAMEA8894903)	N.F2_152	ERS6578072(SAMEA8895163)
S. habrochaites	2019	Peru	Canta	-11.457461	-76.626274	3	S3_Plant4	healthy	G.B5_153	ERS6577553(SAMEA8894643)	N.P9_153	ERS6577813(SAMEA8894904)	N.F2_153	ERS6578073(SAMEA8895164)
